# The Impact of Draping Effects on the Stiffness and Failure Behavior of Unidirectional Non-Crimp Fabric Fiber Reinforced Composites

**DOI:** 10.3390/ma13132959

**Published:** 2020-07-02

**Authors:** Eckart Kunze, Siegfried Galkin, Robert Böhm, Maik Gude, Luise Kärger

**Affiliations:** 1 Institute of Lightweight Engineering and Polymer Technology, Technical University Dresden, Holbeinstrasse 3, D-01307 Dresden, Germany; maik.gude@tu-dresden.de; 2 Karlsruhe Institute of Technology (KIT), Institute of Vehicle System Technology (FAST), Lightweight Technology (LBT), Rintheimer Querallee 2, D-76131 Karlsruhe, Germany; siegfried.galkin@kit.edu; 3 Faculty of Engineering, Leipzig University of Applied Sciences, Karl-Liebknecht-Straße 134, 04277 Leipzig, Germany; robert.boehm.1@htwk-leipzig.de

**Keywords:** unidirectional non-crimp fabrics, draping effects, deformation modes, mechanical properties, fiber volume content, waviness, inter fiber failure, failure envelopes, gapping, shearing

## Abstract

Unidirectional non-crimp fabrics (UD-NCF) are often used to exploit the lightweight potential of continuous fiber reinforced plastics (CoFRP). During the draping process, the UD-NCF fabric can undergo large deformations that alter the local fiber orientation, the local fiber volume content (FVC) and create local fiber waviness. Especially the FVC is affected and has a large impact on the mechanical properties. This impact, resulting from different deformation modes during draping, is in general not considered in composite design processes. To analyze the impact of different draping effects on the mechanical properties and the failure behavior of UD-NCF composites, experimental results of reference laminates are compared to the results of laminates with specifically induced draping effects, such as non-constant FVC and fiber waviness. Furthermore, an analytical model to predict the failure strengths of UD laminates with in-plane waviness is introduced. The resulting stiffness and strength values for different FVC or amplitude to wavelength configurations are presented and discussed. In addition, failure envelopes based on the PUCK failure criterion for each draping effect are derived, which show a clear specific impact on the mechanical properties. The findings suggest that each draping effect leads to a “new fabric” type. Additionally, analytical models are introduced and the experimental results are compared to the predictions. Results indicate that the models provide reliable predictions for each draping effect. Recommendations regarding necessary tests to consider each draping effect are presented. As a further prospect the resulting stiffness and strength values for each draping effect can be used for a more accurate prediction of the structural performance of CoFRP parts.

## 1. Introduction

**Figure 1:**
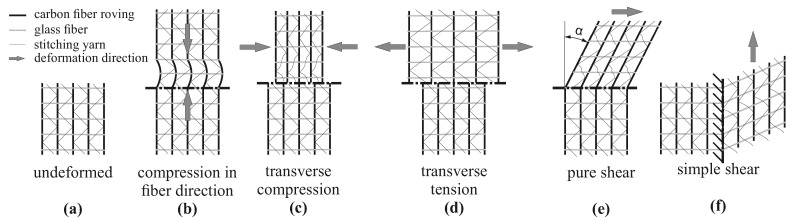
Schematic representation of different deformation modes of a dry unidirectional non-crimp fabric (UD-NCF) [7].

One of the main challenges in the design of structural components using fiber reinforced plastics is the impact of the manufacturing process on the resulting structural performance [1,2]. With increasing part complexity the material behavior is highly affected by the resulting local fiber orientation, local fiber volume content (FVC) and also by local fiber waviness. While continuous fiber reinforced plastics (CoFRP) provide excellent weight-specific stiffness and strength, the material properties must be precisely known in order to exploit their full potential of straight and continuous fibers. When manufacturing CoFRP parts, the dry fabric material can undergo large deformations during the draping process [3]. The degree of the deformation is defined by the composition of the fabric itself. While forming simulations on a macroscopic level provides reliable results regarding the local fabric deformations [4], the actual material properties of the composite are defined after the infiltration step as soon as the laminate is fully cured. As shown in a previous study [5], fabric deformations have a strong impact on the resulting FVC. Although it is possible to manipulate the fabric deformation by specific manufacturing process boundary conditions [6,7,8], a homogeneous distribution of the FVC, without any local gapping or fiber waviness, is difficult to achieve. One of the commonly used fabric types for the production of CoFRP parts are unidirectional non-crimp fabrics (UD-NCF) due to their straight fibers without inherent undulations. This fabric type typically undergoes several deformation modes (cf. Figure 1).

These deformation modes can be clustered in two categories: deformation of the rovings (Figure 1b) and deformation of the stitching yarn (Figure 1c–f). For instance, compression of rovings in fiber direction (Figure 1b), which may result e.g., from run length differences of two adjoining rovings due to deformation in double-curved areas, leads to local fiber waviness. Such deformation modes significantly decrease the stiffness in fiber direction [9,10]. While the material properties of composites with imposed waviness have been widely evaluated [10,11,12,13,14,15,16,17], the impact of the FVC change has not been evaluated yet. In addition, the resulting strength in waviness direction was in most cases analyzed by compressive loads only. Tensile tests on samples with waviness are rare [12,13,16] and usually deal with prepreg fabric material, which has a different deformation modes compared to UD-NCF materials. Especially, a systematical analysis of the resulting strength for tensile and compressive loads in conjunction with analytical predictions is not yet available. Furthermore, most studies deal with out-of plane waviness [10,11,12,13,14,15,16,17], while a focus on in-plane waviness [16,17] is also rare.

In addition to fiber waviness, also the FVC has a considerable impact on the mechanical properties [18]. The main factor that defines the FVC is the areal weight of the fabric. While a target fabric weight is given by the manufacturer, this property can be manipulated by the deformation of the fabric. The deformation modes transverse compression and pure shear (Figure 1c,e) increase the FVC, while transverse tension (Figure 1d) decreases the FVC. The other factor that defines the FVC is the laminate thickness, respectively the cavity height of the tool. In general, only the thickness of the laminate is adjusted to define the FVC and the resulting material properties. However, based on the given deformation modes in Figure 1c–e, the draping process is most likely to cause further increase or decrease of the local areal weight and thus of the FVC. Of all the illustrated deformation modes (Figure 1), only the simple shear mode (Figure 1f) does not have an impact on the resulting FVC, since the rovings can slide along each other. While the impact of FVC on material properties in fiber direction can be easily determined by applying a parallel-connected model [19,20,21], the properties in transverse fiber direction need to be determined by coupon tests. There have been few experimental studies in which the resulting material stiffness is analyzed by a variation of FVC [19,21,22,23]. The resulting transverse strength values and the in-plane shear strength have been analyzed either by adjusting the laminate thickness or the areal weight of the fabric [19,22,24,25]. An investigation of the deformation of the fabric, the associated change of FVC and the resulting strength is not yet available. Further, in case of multiaxial stress states, the impact of FVC on the failure behavior of CoFRPs still needs to be evaluated. For this purpose, existing failure criteria and damage models can be utilized to analyze the resulting failure envelopes [26].

In addition to the previously published studies on draping effects, the goal of this study is to evaluate the impact of fiber volume content changes due to draping effects. To extend the understanding of the resulting material properties with varying FVC and fiber waviness, several preforms are created with and without predefined draping effects (Section 2). To impose the different deformation modes on the fabric, previously created tools are used [5]. To compare each deformation mode with the other, the FVC is defined as the common ground. The FVC is varied in two different ways—by the draping effects gapping and fiber shearing. Additionally, the impact of waviness on the resulting stiffness and strength is compared to straight rovings. To incorporate the experimental results into the modeling and design of composite parts, analytical approaches for stiffness, strength and failure models are presented. The experimental results and the derived observations are given in Section 3 and Section 5.

## 2. Materials and Experimental Methods

### 2.1. Materials

Consistency in transferring results from previous work along a virtual process chain [8,27] and practical relevance were taken into account while selecting materials for this study. For this reason, materials (Table 1) were selected that are commercially available and that are suitable for use in series production, for example in the automotive industry.

**Figure 2:**
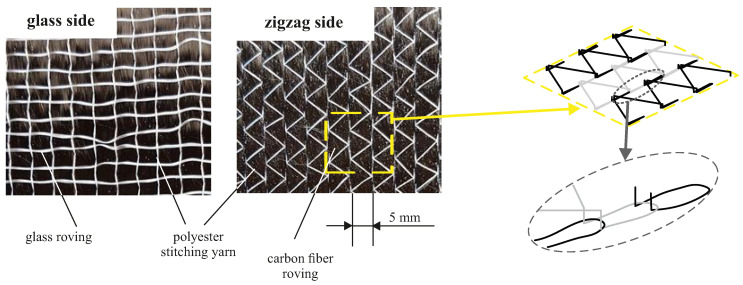
Zoltek PX35 unidirectional non-crimped carbon fiber fabric.

A unidirectional warp knitted non-crimp fabric (UD-NCF) from Zoltek with a measured total areal weight of 338 g m^−2^, comprising the PX35 50 K continuous tow carbon fiber roving was used for this study. The fabric comprises four components: the 5 mm wide carbon rovings, a polyester stitching yarn, thin glass fiber rovings and a factory pre-applied powder binder. Adjacent carbon rovings are connected by a tricot loop type stitching that forms a characteristic zigzag pattern on one side of the fabric (referred to as “zigzag side”, see Figure 2) and gives the fabric a high shearing resistance. On the other side of the fabric, 34 dtex glass rovings are oriented in transverse direction to the carbon rovings that are sewn into the tricot loops for structural integrity of the NCF. The side with the glass rovings is referred to as the “glass side” (cf. Figure 2).

A non-reactive bisphenol-A based epoxy binder, XB 3366 from Huntsman, is factory pre-applied on the zigzag side of the fabric. Binder technology is used to freeze the draping effects after preforming and to assemble subpreforms to a final preform before injection. “Non-reactive” means the binder can be transformed from a solid to a viscous state several times. The softening point of the binder is at 150 °C and it should not be subjected to 200 °C longer than two minutes in circulating air and to 190 °C longer than 25 s in direct contact.

The epoxy resin system Biresin CR170/CH150-3 from Sika was selected for this study due to its low viscosity and reactivity at high temperatures. It is a bisphenol-A based resin in combination with an amine hardener. The material is particularly suitable for injection processes, like high-pressure resin transfer molding, and allows for short cycle times that are required in the automotive industry. It has a glass transition temperature of up to 143 ${}^{{}^{\circ}}C$. Resin viscosity was characterized in [28]. For releasing the parts from the mold, the internal mold release agent PAT 657 BW from Würtz was added to the resin.

Furthermore, the use of an additional reactive binder became necessary in certain areas, where preformed draping effects need to be preserved. For that purpose, the reactive binder XB 6078 from Huntsman is used, since it does not re-soften once it is activated. The application is necessary, because the non-reactive binder re-softens at tool temperature and does not fix the draping effects during injection.

### 2.2. Fiber Volume Content Resulting from Draping Effects

The common reference value for comparing different types of draping effects is the resulting local fiber volume content of the laminate. There are several approaches to determine the resulting FVC for each draping effect [5]. The FVC can be varied either by the areal weight of the fabric or by the thickness of the laminate. The areal weight can be adjusted by a deformation of the fabric, which reduces or increases the roving distance in the observed area. The laminate thickness is adjusted in this work by a local change of the cavity height. In general the FVC φ can be deduced using the following equation

(1) $$\varphi =\frac{n_{L}m_{A_{0}}}{\rho_{f}t}$$

where $n_{L}$ corresponds to the number of plies of the laminate, $m_{A_{0}}$ is the areal weight of the undeformed fabric, $\rho_{f}$ is the density of the fiber and *t* defines the laminate thickness. In the present study the reference samples without draping effects consider only the change of the local laminate thickness, while the fabric itself remains undeformed. Due to scatter of each parameter itself in Equation (1), the resulting FVC is considered to be an approximation.

One possible draping effect is gapping between rovings due to an applied transverse tension to the fabric. Since a gap through the whole laminate would create a weak spot, the evaluated samples consist of a fixation ply and a subpreformed ply with gaps (cf. Table 4 gapping and its stacking). The corresponding FVC can then be estimated by

(2) $$\varphi_{g,stack}=\frac{1}{n_{L}}\left(\left(n_{L}-n_{g}\right)\varphi_{f}+n_{g}\varphi_{g}\left(w_{\mathrm{gap}}\right)\right)=\frac{n_{L}m_{A_{0}}}{\rho_{f}t}\left(1-\frac{n_{g}}{n_{L}}\left(1+\frac{w_{n}}{w_{n}+w_{\mathrm{gap}}}\right)\right),$$

where $n_{g}$ equals to the number of gapped plies, $w_{n}$ corresponds to the width of a roving and $w_{gap}$ defines the gap width. Such an approach combines the FVC from each ply to determine the FVC of the whole stack. It is obvious that gaps can only reduce the FVC of the laminate. By selecting the corresponding gap width, the desired FVC can be adjusted.

One of the major deformation modes of UD-NCF fabrics is fiber shearing that causes transverse compression of the rovings. While for simple shear loads the rovings just slide parallel to each other and the stitching is stretched, for pure shear the rovings are pushed against each other. In such a case, the amount of fibers is increased in the evaluated area, while the cavity height or the resulting laminate thickness remains constant. This condition leads to an increase of the local FVC and can be estimated by the following relationship

(3) $$\varphi_{s}=\frac{n_{L}m_{A_{0}}}{\rho_{f}t}\frac{A_{0}}{A(\alpha )}=\frac{n_{L}m_{A_{0}}}{\rho_{f}t}\frac{1}{\cos\alpha },$$

where $A_{0}$ references to the initial size of the fabric area and A(α) corresponds to the deformed area (cf. Table 4 fiber shearing and its principle of preparation). Contrary to gapping, a pure shear deformation causes an increase of the local FVC within the evaluated area.

Due to deformation of the fabric in double curved areas of a component geometry, a run length difference of two adjoining rovings can occur. Such a case and a local roving compression lead inevitably to local fiber waviness. If the ratio of the waved roving length to the initial roving length of an observed area is known, the local FVC can be estimated by

(4) $$\varphi_{w}=\frac{n_{L}m_{A_{0}}}{\rho_{f}t}\frac{l_{\lambda ,A}}{l_{0}}$$

where $l_{\lambda ,A}$ corresponds to the arc length of the deformed roving, which depends on the amplitude *A* and the wavelength λ, while $l_{0}$ is the initial roving length. Similar to a pure shear load on the fabric, the FVC can only increase due to present waviness, if the thickness stays constant. These analytical approaches have been compared in previous work with experimental results and showed a very good agreement [5]. Therefore, these approaches are used in the present work to determine the quantities of each draping effect so that the same resulting FVC is achieved in the differently prepared laminates. The goal is to compare the impacts of the draping effects on the mechanical properties with each other and with regard to the undeformed reference samples with the same predefined FVC.

#### Design of Experiments

**Figure 3:**
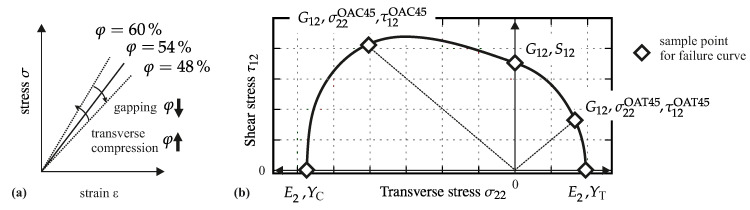
Schematic illustration of the influence of draping effects on mechanical properties (**a**) and visualization of parameters for obtaining sample points for failure curves (**b**).

The basic material parameters that are needed to characterize a transversely isotropic material under in-plane loading conditions are the moduli $E_{1}$, $E_{2}$ and $G_{12}$, the Poisson’s ratio $\nu_{12}$, the fiber strengths $X_{T}$ and $X_{C}$, the transverse strengths $Y_{T}$ and $Y_{C}$ and also the in-plane shear strength $S_{12}$. To determine the influence of draping effects on the stiffness and strength of UD-NCF composites, the experiments are grouped into two major parts, each containing reference samples and samples with draping effects:

Part 1 concerns the influence of the effects gapping and fiber shearing on the mechanical properties transverse to the fiber direction (see Table 2). With these two effects, the rovings are always straight and therefore no impact on the properties in fiber direction is expected. To evaluate the draping effect’s influence on material parameters, the experiments are designed to deliver sample points for deriving a failure envelope (see Figure 3b). This approach enables the comparison of the failure envelopes for different effects with each other and with regard to the reference. Therefore, the parameters listed in Table 2 (also marked in Figure 3b) will be determined for reference samples with fiber volume contents of 48%, 54% and 60%. The number of plies is kept constant at $n_{L}=6$ and the FVC will be varied through changing the thickness of the laminate (cavity height). The corresponding FVC due to gapping is adjusted through different gap sizes between the individual rovings at a constant laminate thickness of 2.25 mm (further details in Section 2.3). The corresponding FVC due to the draping effect fiber shearing is induced by shearing the fabric (shear angle in Table 2). The corresponding gap sizes and shear angles can be calculated, if the FVC is defined and Equations (2) and (3) are solved for the required parameters $w_{g}$ and α. To calculate the gap width, the roving width $w_{n}=5$ mm is used.

Part 2 (see Table 3) concerns the influence of fiber waviness on the basic material parameters in fiber direction, because waviness represents a deviation from the straight fiber orientation. In correspondence to Part 1, the reference samples have a FVC of 48%, 54% and 60%. Two different amplitude to wave length ratios A/λ at a base FVC of 54% (t=2 mm) are investigated.

**Figure 4:**
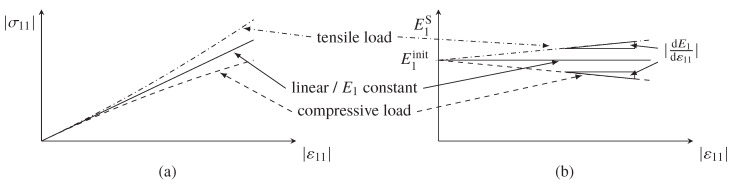
Schematic representation of the stress–strain curves for loads in fiber direction (**a**) and the principle to determine the slope $dE_{1}\;/\;d\varepsilon_{11}$ and the static modulus $E_{1}^{\mathrm{init}}$ from the stress–strain curves (**b**).

Young’s Modulus $E_{1}$ and fiber direction strength values ($X_{T}$ and $X_{C}$) are measured in tension and compression tests. However, in the case of carbon fibers further parameters are needed. If a load is applied in fiber direction, the misorientation of the crystallites within the fibers leads to an increase or decrease of the modulus $E_{1}$ due to reorientation of these crystallites under tensile and compressive strain, respectively [29,30,31,32]. Since the stress in fiber direction $\sigma_{11}$ is directly dependent on the modulus $E_{1}$, the acting strain in fiber direction $\varepsilon_{11}$ is used as the free parameter to define the current modulus. In most cases, the increase of the modulus over the strain can be assumed to be linear. To determine the increase of the modulus and the corresponding static modulus, the secant modulus $E_{1}^{S}=\sigma_{11}\;/\;\varepsilon_{11}$ is plotted against the acting strain. The resulting slope $dE_{1}\;/\;d\varepsilon_{11}$ defines the increase or decrease of the modulus, which sign depends on the loading direction and the intersection with the secant modulus axis corresponds to the initial static modulus $E_{1}^{\mathrm{init}}$. A visualization of this material behavior is given in Figure 4.

These parameters can be easily determined from tensile tests in fiber direction, while special test equipment is needed to determine these parameters from compression tests [29], since the resulting strain tends to be underestimated due to restrained deformations in thickness direction. By considering this fact, all further stiffness values, such as $E_{2}$ and $G_{12}$, are determined from tensile tests. If compressive tests are used to determine the material stiffness, it is recommended to use the strain field on the thickness side of the sample to determine the stiffness. Using the tensile specimen in fiber direction, the Poisson’s ratio $\nu_{12}$ is additionally investigated.

### 2.3. Preforming

The preforming process includes automated cutting of the plies, creating draping effects, stacking of the plies and binder activation. In reference samples no draping effects are induced. After cutting the plies to the final size, six plies are symmetrically stacked, with the zigzag side (comprising the factory applied binder) always facing inwards. The side containing the binder should not make contact with the tool surface, as this makes demolding more difficult. The binder is activated by placing the stack in a membrane press at 100 mbar absolute pressure for 10 min at 155 °C. The activation procedure is the same for all preforms, including preforms with draping effects. Draping effects gapping and waviness are individually induced in each ply, whereas fiber shearing is induced at once in all plies of the stack. The tools and process steps are described in more detail in [5,7]. However a brief description of how the draping effects are created is given.

To induce gaps, a sliding mechanism is used, where the fabric is fixed upon needle ledges (tooling and process principle is shown in Table 4). Each needle ledge has needles arranged one behind another at 5 mm intervals over a distance of 450 mm. The fabric is pressed onto the needles in a way that the needles precisely pierce between the rovings. A spacer bar is then inserted between the needle ledges that creates a transverse tension and thus causes gapping. The width of the spacer bar corresponds to the gap size in Table 2. Ten spacer bars are inserted in the middle of blank fabric sheets with a size of 420 mm×430 mm in a way that a gap according to Table 2 is created over the length of the blank. Since a single ply preform with gaps is not self-supporting and would lose its shape in spite of the binder, an additional ungapped fixation ply is necessary. To achieve the required FVC the gap needs to compensate for the fibers in the fixation ply (see Equation (2)). For gap size $w_{g}=3.3\;mm$, the forming limit of the fabric is reached. Therefore, single roving strands had to be cut from the fabric and placed individually at a distance of 3.3 mm with the help of the needle ledges. The binder in these two-ply subpreforms (see Table 4) is activated with a hot air gun before the subpreform is removed from the sliding mechanism. In order to maintain the symmetrical layer structure for gapped preforms, a seventh ply (undeformed fixation ply) is added to the final stack exclusively in the gapped area. The non-reactive binder is then activated in the same way as the reference stacks and cut to size of the cavity after activation.

The effect fiber shearing is created by placing six oversized sheets of fabric in a shear frame and shearing the frame by the specific angle according to Table 2. To fix the shear deformation, the sheared fabric is placed with the shear frame into the membrane press and activated according to the parameters previously mentioned. Afterwards it is cut to the size of the cavity.

Waviness is also induced with a sliding mechanism where the fabric is pressed onto needle ledges. However, in this case, the needle ledges are oriented perpendicular to the fiber direction (see Table 4). By pushing every other needle ledge into equally opposing directions up to five periods of the wavelength λ=20 mm with different amplitudes *A* can be adjusted. The needle ledges are pushed simultaneously with the help of so-called master plates. The pattern machined into the master plates determines the amplitudes and by this the amplitude to wavelength ratio A/λ can be adjusted. For this study A/λ ratios of 0.03 over five periods and 0.06 over three periods are set. In [5] further details setting the FVC for the draping effect waviness can be found. Waviness in the deformed ply is fixed by activating the binder with a hot air gun while the fabric is still pressed onto the needle ledges. After removal from the tool, six plies are symmetrically stacked, adding 5 g m^−2^ to 8 g m^−2^ of the reactive binder XB 6078 from Huntsman. The additional binder is necessary since first impregnated samples showed that the factory pre-applied non-reactive binder does not securely fix the draped waviness at tool temperature. During injection the amplitude was changed due to fiber washout. Reactive binder is only added onto the area with the draping effect waviness and 3 cm beyond. The stacked plies are activated in a membrane press with the same parameters as previously defined. The whole stack is cut to the size of the cavity afterwards.

### 2.4. Resin Transfer Molding and Post Cure

**Figure 5:**
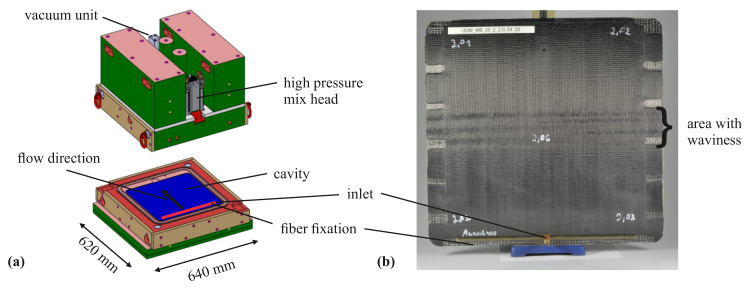
Resin-transfer molding tool (**a**) and plate with imposed fiber waviness with an A/λ=0.06 ratio (**b**).

The preforms are impregnated in a high-pressure resin transfer molding process. Table 5 lists the process parameters. The plate tool in Figure 5 in combination with a high-pressure metering machine EPOxMix from FRIMO is used. This process is required due to the reactivity and viscosity of the epoxy resin system and the large number of preforms (in total more than 35 preforms). The stiff tool has interchangeable inserts (blue region in Figure 5a) for adjusting plate thickness. With respect to the overall plate thickness, a standard deviation of 0.022 mm is achieved, which is important in adjusting a precise FVC. The resin flow during mold filling is along the direction of the fibers over the entire width of the plate. The tool has a vacuum gate that can be closed before injection. To accomplish a laminate without pores, the tool is evacuated for 2 min prior to injection to achieve an absolute pressure in the cavity of 1.3 mbar to 2.0 mbar. For careful mold filling, the resin is injected at a mass flow rate of 10 g s^−1^. Depending on the plate thickness and FVC, the injected resin mass varies between 150 g to 220 g. To ensure optimal plate quality, the process is carried out in such a way that cavity pressures of 50 bar to 60 bar are achieved at the end of injection. Cavity pressure is monitored with type 4001A sensors from Kistler. It was found that the non-reactive binder could not securely fix the preform in the plate tool, resulting in deviations from the straight fiber orientation. To prevent the deformation of fibers during injection, a 2 mm thick silicone strip is placed on the preform between the inlet gate and the edge of the preform (mark at the bottom of plate in Figure 5b) to act as a fiber clamping. The plate is cured 13 min at 100 ${}^{{}^{\circ}}C$ tool temperature. Demolding behavior is excellent due to the internal mold release agent. Post curing was carried out in a convection oven for several plates together.

### 2.5. Specimen Preparation

Sections that already contain all test specimens in the correct length are separated from the RTM plates by means of a water jet cutting. On both sides of these plates 1 mm thick, glass/epoxy FRP tabs with a fiber orientation of ±45° are glued onto the end areas. The adhesive DP490 from Scotch Weld is used. Strip-shaped samples are then cut off with a water-cooled diamond cutting disc using a Axitom automatic cutting machine from Struers. This procedure has the advantage that the edges of the specimen are absolutely smooth and perpendicular to the specimen surface. Furthermore, the edges of the tabs correspond exactly to the edges of the specimen. For off-axis tension specimen oblique tabs were applied. In [33,34,35] it is found that oblique tabs reduce stress concentrations leading to a more uniform strain field within the off-axis specimen.

### 2.6. Mechanical Testing and Experimental Analysis

The performed tests, the standards, the test equipment used and the size of the specimens to fulfill the design of experiments presented in Section 2.2 are listed in Table 6 and Table 7. A constant strain rate of $1\times 10^{-4}\;S^{-1}$ is applied in all tests to achieve comparable material behavior for all types of tests.

This is realized by setting the test speed according to the free length of the specimen for each type of test in Table 6 and Table 7. All tests are carried out under standard climate conditions (23 ${}^{{}^{\circ}}C$ and 50% relative humidity). A minimum of five specimens is tested for each type of test and each fiber volume content. Strain measurement for all samples with draping effects and off-axis specimens is realized by the optical strain measurement system Aramis from GOM. This approach allows for in-situ full field strain measurement and monitoring of the deformations in the areas with draping effects. Thus, the failure behavior can be analyzed in detail. Furthermore, for reference off-axis tension and compression tests, this method is very useful to directly measure the strain in fiber direction and transverse to it. For the other reference specimens, contactless video and laser extensometer strain measurement is applied.

At the edge of specimens with waviness, the load path in the undulated fibers is interrupted by cutting the specimen to size. For this reason, the width was increased compared to the reference samples in order to have several continuously undulated rovings running in the center of the specimen. Testing specimens with waviness in compression is more complex. To avoid off-axis failure when the free length of the specimen is only 10 mm (according to the standard DIN ISO 14126), the free length of the compressive test specimens had to be increased to 2λ, corresponding to a free length of 40 mm. To prevent premature buckling a support block is used.

In general, in many standards, the stiffness is evaluated by calculating the slope between two specific strain points. However, the DIN EN ISO 527 standard suggests to use either the two point method to determine the secant stiffness or to use a regression which returns the tangent modulus. If the secant stiffness is used as input for structural simulation, deviations between experimental and numerical results can occur due to noisy measurement or due to positioning of the points at nonlinear material behavior. Therefore, the regression method has been used instead to compare the results of different tests and for different standards. In contrast to the suggested evaluation range ε=[0.0005,0.0025] of the regression, the least square error was determined over the whole strain range and the strain range with the highest $R^{2}$ value was used.

## 3. Experimental Results

### 3.1. Reference Samples

The resulting material properties for reference samples at the three different FVCs of 48%, 54% and 60% are summarized in Table 8 and Table 9 with median values and the corresponding interquartile range.

#### 3.1.1. Material Stiffness Properties of Reference Samples and their Fiber Volume Content Dependency

**Figure 6:**
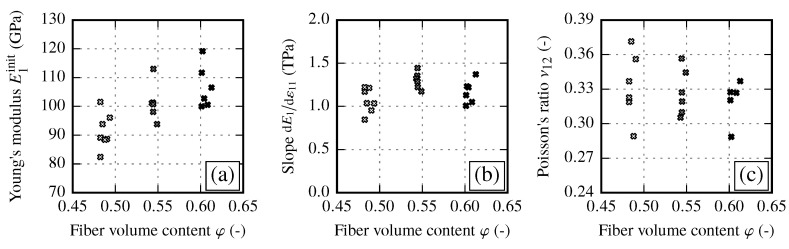
Reference samples—material parameters in fiber direction vs. the fiber volume content: Static modulus $E_{1}^{\mathrm{init}}$ (**a**), the constant slope $dE_{1}\;/\;d\varepsilon_{11}$ to determine the resulting modulus $E_{1}$ at arbitrary $\varepsilon_{11}$ values (**b**) and the Poisson’s ratio $\nu_{12}$ (**c**).

As expected, longitudinal Young’s modulus $E_{1}$ in fiber direction increases with increasing fiber volume content. Figure 6a shows the initial static modulus $E_{1}^{\mathrm{init}}$, which is determined at the very beginning of loading according to the method described at the end of Section 2.2. The curve was linearly fitted in a strain range, where the slope is nearly constant. In contrast to the Young’s modulus according to the DIN EN ISO 527-5 standard, which is determined in a strain range ε=[0.0005,0.0025], $E_{1}^{\mathrm{init}}$ is about 3% lower. Figure 6b shows the increase of the Young’s modulus $E_{1}$ over the strain, which is represented by the slope $dE_{1}\;/\;d\varepsilon_{11}$ of the linear fit. Since the increase of $E_{1}$ depends on the carbon fiber itself, the slope $dE_{1}\;/\;d\varepsilon_{11}$ should also increase with increasing FVC. However, from the analyzed results no obvious increase is observed. Instead, the increase of $E_{1}$ seems to be independent of the FVC. One possible explanation is the fact that the fabric has an material specific waviness due to the glass fibers and the sewing. To develop a pronounced increase of $E_{1}$, such waviness must be resolved due to stretching of the roving. The Poisson’s ratio $\nu_{12}$ shows a high scatter for lower FVC values. By applying a parallel-connected model, the Poisson’s ratio should decrease with increasing FVC. However, due to large scatter, such pronounced behavior is not observed. A slightly decreasing trend with increasing FVC can be noted, as the spread clearly reduces at higher FVC.

**Figure 7:**
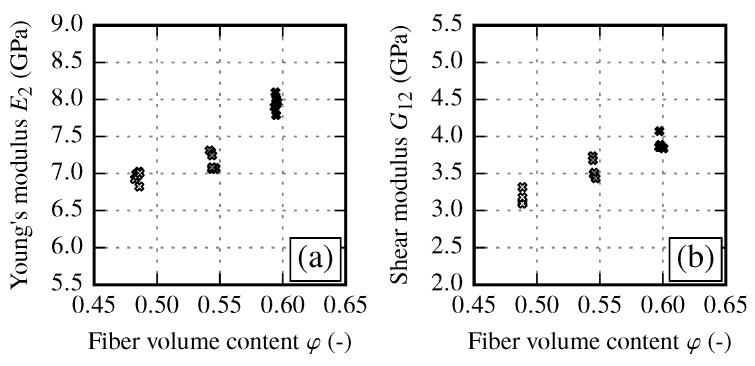
Reference samples—transverse stiffness $E_{2}$ (**a**) and shear modulus $G_{12}$ (**b**) vs. the fiber volume content.

The transverse Young’s modulus $E_{2}$ in fiber transverse direction and shear modulus $G_{12}$ clearly increase with an increasing FVC (Figure 7). Such a behavior is expected since the transverse stiffness and the shear modulus of the fiber is much higher as of the matrix. In should be noted, the shear modulus values obtained from off-axis tensile tests (Figure 7b) show less scatter than the comparable values from V-notch rail shear tests according to ASTM 7078 (not shown). Therefore, ASTM 7078 results are used for evaluation of shear strength $S_{12}$ only.

#### 3.1.2. Strength Properties of Reference Samples and their Fiber Volume Content Dependency

**Figure 8:**
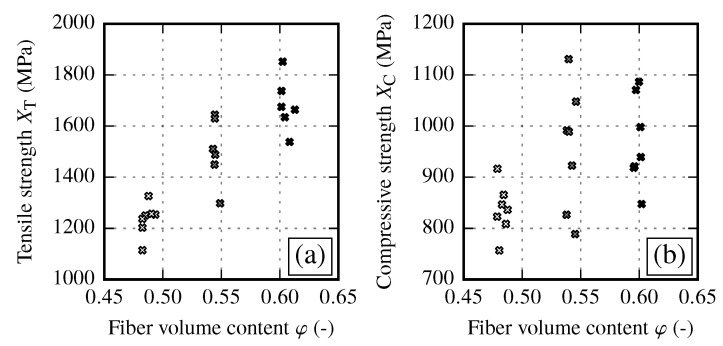
Reference samples—tensile and compressive strength in fiber direction $X_{T}$ (**a**) and $X_{C}$ (**b**) vs. different fiber volume content values.

Tensile and compressive strength of reference samples are determined for the same three FVCs as the stiffness properties. In fiber direction, a clear dependency of the tensile strengths on the FVC is present (Figure 8a). For compression loads (Figure 8b), the spread is found to be larger compared to the tensile tests. Yet, a clear increase of the compressive strength from φ≈48% to φ≈54% can be seen. Due to the spread of the test results, the tendency can be assumed for the step from φ≈54% to φ≈60%, however it is not represented be the median values (Table 9).

**Figure 9:**
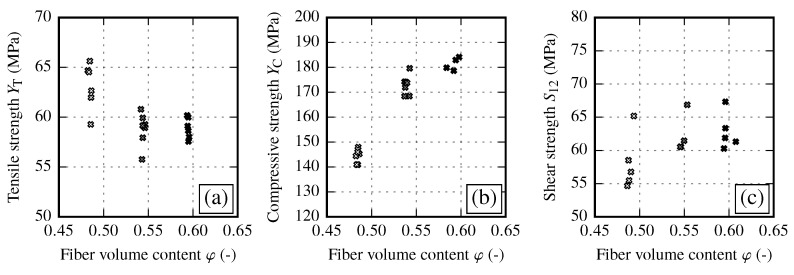
Reference samples—tensile and compressive strength in transverse direction $Y_{T}$ (**a**) and $Y_{C}$ (**b**); and shear strength $S_{12}$ (**c**) from V-notch rail shear tests according to ASTM 7078 vs. the fiber volume content.

In transverse fiber direction, material properties are dominated by the matrix of the composite. With increasing FVC, the resulting transverse tensile strength $Y_{T}$ is slightly reduced (Figure 9a). Shear strength shows a sligtht trend towards higher values for increasing FVC (Figure 9c). In the case of fiber shearing, the strength increases significantly from φ≈48% to φ≈54%. However, if the FVC is further increased the resulting strength seems to run into saturation (Figure 9b). Compared to other strength parameters, the transverse compressive strength shows the most distinct sensitivity against the FVC. This can result from the fact that for compressive loads, local material imperfections do not lead to a sudden failure as it is for tensile loads.

### 3.2. Samples with Draping Effects

#### 3.2.1. Gapping

**Figure 10:**
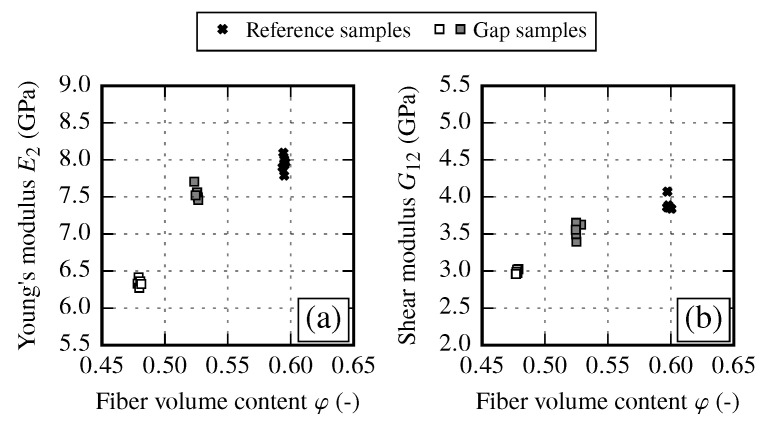
Gapping samples—resulting material stiffness in transverse direction (**a**) and shear modulus (**b**) from off-axis tensions tests (OAT45).

Gapping samples are prepared by transversely stretching the fabric and thus reducing the fiber volume content (see Section 2.3). The resulting material properties are compared to the initial state, which is the reference sample at φ≈60%. The stiffness properties of gapping samples show a very low spread at different fiber volume contents. As expected, the FVC is reduced due to gaps. Therefore, the transverse modulus $E_{2}$ as well as the shear modulus $G_{12}$ decrease (Figure 10 and Table 10). The modulus $E_{2}$ at φ≈54% and φ≈48% is reduced by 6% and 20% compared to the reference samples at φ≈60%, while the shear modulus $G_{12}$ decreases by 8% and 22.5% for the corresponding FVC. Gapping has an impact on both stiffness parameters in the same way.

**Figure 11:**
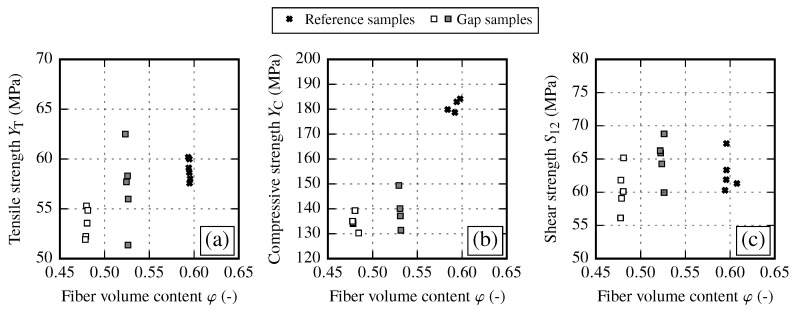
Gapping samples—tensile and compressive strength in transverse direction $Y_{T}$ (**a**) and $Y_{C}$ (**b**); and shear strength $S_{12}$ (**c**) from V-notch rail shear tests according to ASTM 7078 at different fiber volume contents.

The determined tensile strength results $Y_{T}$ at different gap sizes are given in Figure 11a. Occuring gaps slightly decrease the tensile strength according to the gap size by 2 (φ≈54%) and 9% (φ≈48%) compared to the initial state reference samples. The results of the compressive test convey a different picture. Independently of the gap size, the compressive strength $Y_{C}$ is strongly reduced by about 25% compared to the initial state (Figure 11b) for both gap sizes and falls below the level of $Y_{C}$ for reference samples at φ≈54% and φ≈48%. This drop in compressive strength could be attributed to the resin rich zones due to gapping of the fabric or due to increased local material imperfections. This conditions could lead to a missing support under compressive loading and therefore to a premature failure. In contrast, gapping seems to have no impact on the shear strength $S_{12}$ (cf. Figure 11c). A summary of the resulting strength values compared to the initial state is given in Table 11.

#### 3.2.2. Fiber Shearing

**Figure 12:**
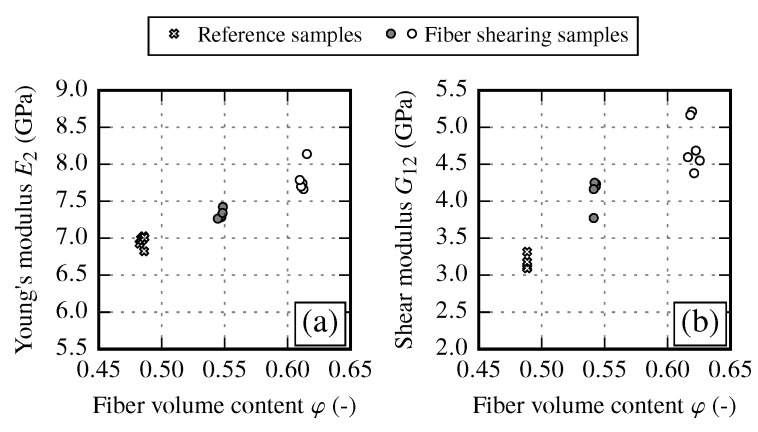
Fiber shearing samples—transverse $E_{2}$ (**a**) and shear modulus $G_{12}$ (**b**) at different fiber volume contents.

Fiber shearing samples are prepared by shearing the fabric and thus increasing the fiber volume content (see Section 2.3). The resulting material properties are assessed in relation to the initial state, which corresponds to the reference samples at φ≈48%. As expected, the transverse modulus $E_{2}$ shows an increase of 4.4% and 11% with increasing FVC compared to the initial state (cf. Figure 12a and Table 12). Shear stiffness $G_{12}$ exhibits a significant increase with increasing shear angle by 33% and 47% compared to the undeformed reference samples (cf. Figure 12b and Table 12). The resulting values are even 20% higher compared to the corresponding samples with the same FVC.

**Figure 13:**
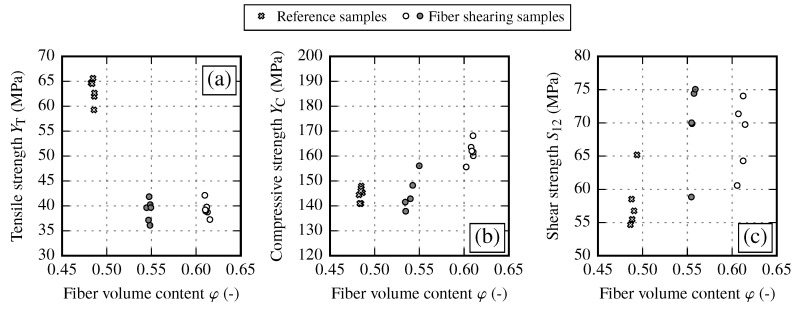
Fiber shearing samples—tensile and compressive strength in transverse direction $Y_{T}$ (**a**) and $Y_{C}$ (**b**); and shear strength $S_{12}$ (**c**) at different fiber volume contents.

One major aspect of samples with fiber shearing is the significant drop of the transverse tensile strength $Y_{T}$ by 38% compared to the initial state (cf. Figure 13a and Table 13). This behavior appears independent of the magnitude of the shearing angle. In contrast, from reference samples a minimal decrease in tensile strength with increasing FVC is observed, but not to such an extend. Possible explanations are discussed in Section 5.4. The influence of fiber shearing on the transverse compressive strength $Y_{C}$ is ambiguous (cf. Figure 13b). Small shear angles do not seem to have an influence on $Y_{C}$. On the other hand for large shear angles, which correspond to φ≈60%, the strength values are increased. Shear strength results show a large spread, yet the trend towards higher values with increasing FVC is clearly visible (cf. Figure 13c). Independent of the shear angle, shear strength $S_{12}$ is increased by 23% compared to the undeformed reference sample at φ≈48%.

### 3.3. Waviness

**Figure 14:**
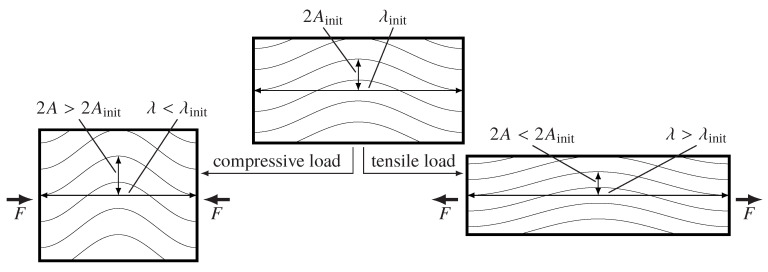
Schematic representation of the initial and deformed state of a wavy section and the corresponding change of the amplitude *A* and the wavelength λ in dependence of the applied load direction.

In addition to the described impact of the draping effects such as gapping or fiber shearing on each material parameter, waviness causes multiple local changes of the material properties. First of all, waviness changes the fiber orientation. As shown in Figure 18, it has also an impact on the resulting FVC, which itself changes the local material properties. A further effect of waviness is the change of the resulting effective stiffness and strength compared to non-undulated areas. Waviness can be defined by its amplitude *A* and wavelength λ. However, if a load is applied these parameters change (see Figure 14).

In case of a uniaxial tensile or compressive load in waviness direction, the amplitude reduces and the wavelength increases for tensile loads and the other way around for compressive loads. Depending on the loading direction, this change of the amplitude to wavelength ratio could lead to a higher risk of premature failure.

**Figure 15:**
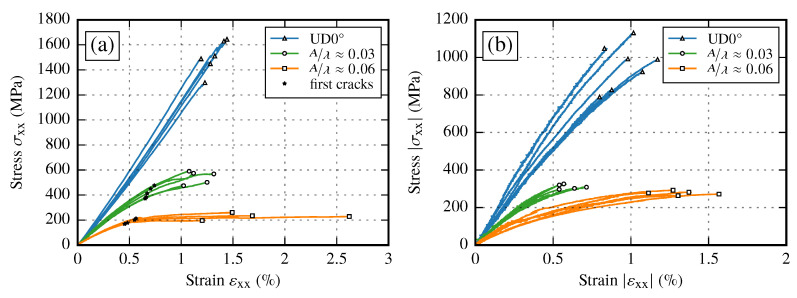
Experimental results of tensile (**a**) and compressive (**b**) tests in comparison to nonundulated UD0° coupon tests.

The experimental results for two different amplitude to wavelength ratios A/λ≈{0.03,0.06} in different loading directions are given in Figure 15. If the resulting stress–strain curves are compared to the results of the UD0° coupon tests (φ≈54%) it can be observed that stiffness and strength are highly affected. The resulting stiffness of the samples with an imposed waviness is independent of the loading direction. The stiffness is reduced by 31% for A/λ≈0.03 and by 58% for A/λ≈0.06 compared to the static modulus $E_{1}^{\mathrm{init}}$. However, the strength shows a large difference. While tensile loads reduce the strength values with increasing A/λ ratios, for compressive loads the strength suddenly drops to an almost equal value, independent of the imposed A/λ ratio. This indicates that the failure modes depend on the loading direction and need to be considered in structural simulation models. Compared to the stiffness drop, the strength values are reduced even more compared to the nonundulated coupon tests (15% to 40% of the tensile strength $X_{T}$ and 28% to 31% of the compressive strength $X_{C}$).

**Figure 16:**
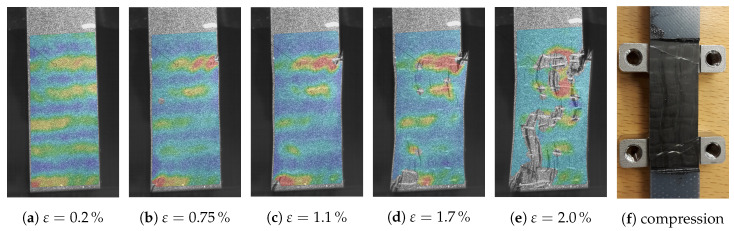
Evolution of deformation under tensile load for an amplitude to wavelength ratio of A/λ≈0.06 (**a**–**e**) and resulting crack after compression for A/λ≈0.03 (**f**).

In case of tensile tests, cracks prior to final failure, first occurring at the edges of the samples, can be observed (analysis of images of full field strain measurement). After the first cracks appear, further cracks are formed within the sample itself. These new cracks occur at the turning points of the wave, which correspond to the positions of the maximum fiber misalignment angle $\theta_{\max}$. If the load is further increased, the cracks start to grow along the fiber direction and an obvious straightening of the fibers can be observed. A visualization of the occurring cracks at different strain states is given in Figure 16a–e. One major observation is the fact that the occurring cracks are more pronounced for higher A/λ ratios, while for smaller values of A/λ final failure occurs shortly after first cracks initiated at the edges. For each sample, the first appearance of a crack is marked in Figure 15. The corresponding stress values lie 18% to 28% below the final failure. This indicates that after first cracks are formed, in contrast to UD laminates, there are still load capabilities of the laminate until final failure available. It should be noted that the occurring cracks could be observed only in tensile tests. For compression tests the specimens have to be stabilized by an additional support to prevent kinking. However, after the compression tests are completed one or two single pronounced cracks over the whole sample could be observed (cf. Figure 16f). All experimental results are summarized in Table 14.

## 4. Analytical Methods for Stiffness and Failure Modeling with Draping Effects

### 4.1. Inter Fiber Failure Criteria for Composites

To evaluate the impact of each draping effect, the experimental results need to be compared to each other. In particular, the inter fiber failure (IFF) evaluates the stresses that cause matrix failure in a composite. There are several approaches to determine a suitable failure envelope for CoFRPs based on the experimentally observed failure stresses of the composite [36,37,38,39]. Although such failure criteria are initially developed for unidirectional fabric, they can also be applied to UD-NCF materials [26]. The PUCK failure criterion uses a physical approach to interpret the IFF of composites and has performed exceptionally well in a broad evaluation of different failure criteria [40,41]. It utilizes the idea of MOHR that fracture of brittle materials, such as composites, is determined by the stresses on the action plane. The action plane is initially unknown and must be iteratively determined. For transversely isotropic materials, the three-dimensional stress state is rotated around the fiber direction and the so called stress exposure $f_{E}$ is calculated for each rotation angle. This factor $f_{E}$ defines the ratio between the current stress and the stress in the same direction at the point of failure. By calculating the stress exposure for each angle, the action plane is the one with the overall highest value $f_{E}$ [42,43]. For in-plane load cases, a two-dimensional formulation of PUCK’s criterion yields

(5) $$f_{E}=\left\{\begin{array}{cc} \sqrt{{\left(\left(\frac{1}{Y_{T}}-\frac{p_{n1}^{t}}{S_{12}}\right)\sigma_{22}\right)}^{2}+{\left(\frac{\tau_{12}}{S_{12}}\right)}^{2}}+\frac{p_{n1}^{t}}{S_{12}}\sigma_{22}, & \sigma_{22}\geq 0\\ \sqrt{{\left(\frac{p_{n1}^{c}}{S_{12}}\sigma_{22}\right)}^{2}+{\left(\frac{\tau_{12}}{S_{12}}\right)}^{2}}+\frac{p_{n1}^{c}}{S_{12}}\sigma_{22},\; & \sigma_{22}<0\wedge |\frac{\sigma_{22}}{\tau_{12}}\left|\leq \right|\frac{S_{23}^{ap}}{\tau_{12}^{c}}|\\ \left({\left(\frac{\tau_{12}}{2\left(1+p_{nt}^{c}\right)S_{12}}\right)}^{2}+{\left(\frac{\sigma_{22}}{Y_{C}}\right)}^{2}\right)\frac{Y_{C}}{\left|\sigma_{22}\right|}, & \sigma_{22}<0\wedge |\frac{\sigma_{22}}{\tau_{12}}\left|\geq \right|\frac{S_{23}^{ap}}{\tau_{12}^{c}}|. \end{array}\right.$$

with

(6) $$S_{23}^{ap}=\frac{Y_{C}}{2\left(1+p_{nt}^{c}\right)}\;\mathrm{and}\;\tau_{12}^{c}=S_{12}\sqrt{1+2p_{nt}^{c}}$$

where $Y_{T}$, $Y_{C}$ and $S_{12}$ are the material strength values and $p_{n1}^{t,c}$, $p_{nt}^{t,c}$ are the so called inclination parameters. In general, the inclination parameters define the slope of the failure surface at the transition points from a tensile (t) to a compressive (c) load. If the inclination parameters are not equal, than a kink in the failure surface is created. For a detailed discussion of the choice of the inclination parameters see [22,43]. For simplicity reasons, the inclination parameters are set to be equal in the present work. Since the material strength values itself are dependent on the FVC, the failure criterion needs to be evaluated for each FVC alone.

### 4.2. Stiffness from Waviness

**Figure 17:**
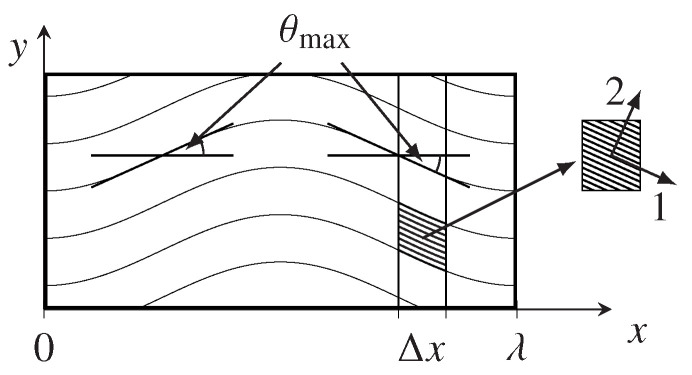
Representative waviness region defined by the amplitude *A* and the wavelength λ.

To determine the effective material properties of areas with waviness, a homogenization step of the local material properties is needed. A representative area can be extracted by assuming a periodic pattern of the waviness. Such a region of interest is given in Figure 17.

Here the waviness is approximated by

(7) $$y=A\sin\frac{2\pi x}{\lambda },$$

where *A* is the amplitude of the curved fiber. To determine the angle θ of the fiber, which corresponds to the fiber direction at *x*, the derivative of *y* to *x* can be utilized

(8) $$\tan\theta =\frac{dy}{dx}=\frac{2\pi A}{\lambda }\cos\frac{2\pi x}{\lambda }.$$

The maximal misalignment angle $\theta_{\max}$ is located at the turning points of the wave (cf. Figure 17) and is defined by amplitude *A* and wavelength λ alone

(9) $$\theta_{\max}=\arctan\frac{2\pi A}{\lambda }.$$

**Figure 18:**
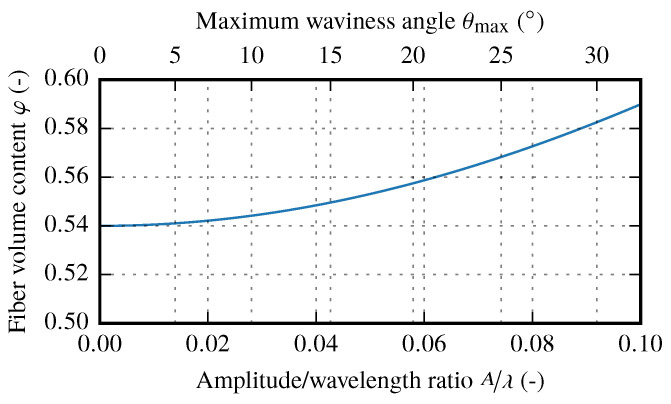
Effect of the amplitude to wavelength ratio A/λ or the corresponding maximal fiber deviation angle $\theta_{\max}$ on the resulting fiber volume content.

As mentioned in Section 2.2, a present waviness increases the local FVC. Since the arc length of a wave $l_{\lambda ,A}$ is a function of *A* and λ, it can be also expressed in terms of the A/λ ratio. In addition with the maximal misalignment angle $\theta_{\max}$ the relation between these two parameters and the FVC can be determined (cf. Figure 18).

To determine the effective material properties of a section with wavy fibers the relationship between global strain $\varepsilon_{x}$ and the resulting global stress $\sigma_{x}$ can be utilized. The material properties of an infinitesimal slice Δx (cf. Figure 17) can be obtained by using the material compliance S of a transversely isotropic material and by rotating the local strain $\varepsilon_{1}$ and stress $\sigma_{1}$ into the global coordinate system. This relationship can be expressed as following

(10) $$\varepsilon_{x}=R^{⊤}\varepsilon_{1}=R^{⊤}S\sigma_{1}=\underset{\bar{S}}{\underset{︸}{R^{⊤}SR}}\sigma_{x}$$

where R is a 6×6 rotation matrix whose components are functions of the angle θ, and $\bar{S}$ is the effective material compliance for the slice Δx. It should be noted that the strains and stresses in the equation above are written in a vector form and the compliance matrix is given by a 6×6 matrix. By replacing the components of the rotation matrix R using Equation (8), the effective compliance $\bar{S}$ can be defined as a function of *x*. To obtain the resulting components of the homogenized compliance matrix $\bar{S}$ not only for a slice Δx, but rather for the whole wavelength λ, each component of the transversely isotropic compliance matrix S must be integrated over the path defined by the wave

(11) $$\bar{S}=\frac{1}{\lambda }\int_{0}^{\lambda }Sdx.$$

Since each component of $\bar{S}$ is a function of the angle θ, the integral is applied only to functions of this angle. For instance ${\bar{S}}_{11}$ yields

(12) $${\bar{S}}_{11}=\cos^{4}\left(\theta \right)S_{11}+\sin^{2}\left(\theta \right)\cos^{2}\left(\theta \right)\left(2S_{12}+S_{44}\right)+\sin^{4}\left(\theta \right)S_{22}.$$

The inverse of ${\bar{S}}_{11}$ can be interpreted as the effective Young’s modulus $E_{x}$ in waviness direction

(13) $$E_{x}=\frac{1}{\Upsilon_{1}S_{11}+\Upsilon_{3}\left(2S_{12}+S_{66}\right)+\Upsilon_{2}S_{22}}$$

with

(14) $$\Upsilon_{1}=\frac{1}{\lambda }\int_{0}^{\lambda }\cos^{4}\theta dx=\frac{1+\frac{1}{2}{\left(2\pi A\;/\;\lambda \right)}^{2}}{{\left(1+{\left(2\pi A\;/\;\lambda \right)}^{2}\right)}^{3\;/\;2}},$$

(15) $$\Upsilon_{2}=\frac{1}{\lambda }\int_{0}^{\lambda }\sin^{4}\theta dx=1-\frac{1+\frac{3}{2}{\left(2\pi A\;/\;\lambda \right)}^{2}}{{\left(1+{\left(2\pi A\;/\;\lambda \right)}^{2}\right)}^{3\;/\;2}},$$

(16) $$\Upsilon_{3}=\frac{1}{\lambda }\int_{0}^{\lambda }\sin^{2}\cos^{2}\theta dx=\frac{\frac{1}{2}{\left(2\pi A\;/\;\lambda \right)}^{2}}{{\left(1+{\left(2\pi A\;/\;\lambda \right)}^{2}\right)}^{3\;/\;2}},$$

and

(17) $$S_{11}=\frac{1}{E_{1}},\;S_{12}=-\frac{\nu_{12}}{E_{1}},\;S_{22}=\frac{1}{E_{2}}\;\mathrm{and}\;S_{66}=\frac{1}{G_{12}}.$$

It is obvious that the effective Young’s modulus $E_{x}$ only depends on the local material properties $E_{1}$, $E_{2}$, $G_{12}$, $\nu_{12}$ and the amplitude to wavelength ratio A/λ. The local material parameters are also functions of the fiber volume content. This condition is especially important since, according to the analytical solution for the fiber volume content of samples with a certain A/λ ratio (Equation (4)), the areal weight increases with increasing A/λ ratio. Therefore, for a given sample thickness, the local material parameters increase with a growing A/λ ratio and affect the analytical solution of the resulting effective stiffness $E_{x}$.

### 4.3. Strength from Waviness

While the resulting strength of non-undulated CoFRPs is widely evaluated, both experimentally and numerically [19,20,21,23,44,45,46,47,48,49], the occurring waviness in composites is in most cases evaluated only for compression loads [10,11,12,13,14]. This is caused by the fact that intrinsic waviness of the unidirectional fabric is the main reason for compression failure. Therefore, advanced failure criteria for compression stress loads in fiber direction consider the intrinsic fiber misalignment [39]. For instance, a micromechanical study by GUTKIN [50] showed that the shear stress $\tau_{12}$ in conjunction with fiber misalignment angle can be expressed by a simple failure criterion for compressive loads in fiber direction

(18) $$f_{C}=\frac{|\sigma_{11}|}{X_{C}}+\frac{|\tau_{12}|}{S_{12}},$$

where $X_{C}$ is the compressive strength in fiber direction and $S_{12}$ is the in-plane shear strength of the composite. For tensile loads, the resulting strength of unidirectional laminates is usually determined from the FVC in conjunction with the matrix and fiber strength values. While it is possible to make use of the mentioned approach in Equation (18) to determine the compressive strength for laminates with an imposed waviness, analytical methods for tensile loads, which consider waviness, are not known. Since an off-axis load case can be assumed at the maximum misalignment angle $\theta_{\max}$, the failure criterion for IFF can be utilized at this point to determine the load capability of laminates with imposed waviness. For an off-axis load case, the global stress $\sigma_{\mathrm{xx}}$ is applied to the evaluated area. This stress can be rotated by utilizing $\theta_{\max}$ to determine the local stresses

(19) $$\begin{array}{cc} \sigma_{11} & =\sigma_{\mathrm{xx}}\cos^{2}\theta_{\max}\\ \sigma_{22} & =\sigma_{\mathrm{xx}}\sin^{2}\theta_{\max}\\ \tau_{12} & =-0.5\sigma_{\mathrm{xx}}\sin2\theta_{\max}. \end{array}$$

If the local stresses $\sigma_{22}$ and $\tau_{12}$ are plugged into Equation (5) and the resulting global stress $\sigma_{\mathrm{xx}}$ is evaluated at $f_{E}\overset{!}{=}1$, an estimation of the strength of the laminate with a present waviness can be determined. However, for small off-axis angles the local stress in fiber direction $\sigma_{11}$ is more dominant than the $\sigma_{22}$ and $\tau_{12}$ stresses. Therefore, if the global stress based on the IFF criterion is determined, the resulting stress can exceed the tensile or compressive strength in fiber direction of straight unidirectional laminates. To avoid this inconvenience, the following case distinction is used

(20) $$X_{T,C}=\min\left\{\sigma_{\mathrm{xx}}^{\mathrm{IFF}},X_{T,C}^{UD0{}^{\circ}}\right\},$$

where $X_{T,C}$ is the resulting laminate strength based on the IFF criterion that considers waviness, $\sigma_{\mathrm{xx}}^{\mathrm{IFF}}$ is the solution of the IFF criterion at $f_{E}\overset{!}{=}1$ and $X_{T,C}^{UD0{}^{\circ}}$ is the material strength in fiber direction, if no waviness is present. In the same manner, the failure criterion for fiber stress dominated compression loads (see Equation (18)) can be solved at the failure point $f_{C}\overset{!}{=}1$ to the global stress $\sigma_{\mathrm{xx}}$ to consider waviness in composites

(21) $$\sigma_{\mathrm{xx}}=\frac{2X_{C}S_{12}}{2S_{12}\cos^{2}\theta_{\max}-X_{C}\sin2\theta_{\max}}.$$

This solution applies only to compression loads, since for tensile loads the failure should occur after first IFF cracks occur.

## 5. Discussion and Evaluation of Draping Effects

### 5.1. Resulting Stiffness and Strength for Different Draping Effects

**Figure 19:**
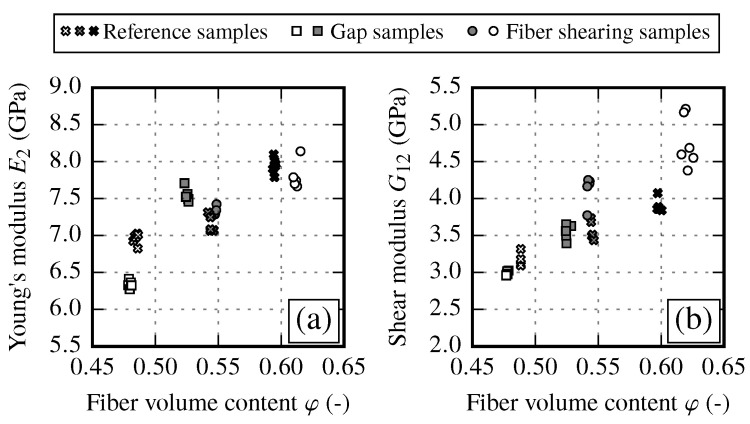
Comparison of the transverse modulus $E_{2}$ (**a**) and the in-plane shear modulus $G_{12}$ (**b**) for different draping effect types.

To compare the mechanical response of each draping effect, the FVC is used as the common factor. By evaluating the resulting stiffness $E_{2}$ and $G_{12}$, an overall increase towards higher FVCs can be observed for all draping effects (cf. Figure 19).

However, each effect has a different impact on the stiffness. While the transverse stiffness $E_{2}$ shows a very good coincidence between reference and fiber shearing samples, the gap samples show a distinct drop of the stiffness (φ≈48%) compared to other results. On the other hand the shear modulus $G_{12}$ shows good agreement between reference and gap samples. It can be observed that fiber shearing samples induce a higher shear stiffness with increasing FVC compared to reference sample results. From the experimental results, it is recommended to use the reference samples configuration (undeformed fabric) to determine the increase of the transverse stiffness. To define the envelope of the shear stiffness over the FVC the use of reference samples configuration is also suitable. However, the results suggest that for very high FVCs (φ>60%) the increase of the shear modulus is more pronounced since the fiber shear stiffness is more dominant than the matrix shear stiffness. Therefore, to get reliable supporting points of the shear stiffness at φ>60% it is recommended to manufacture samples with a FVC φ≈ 62% to 65%.

**Figure 20:**
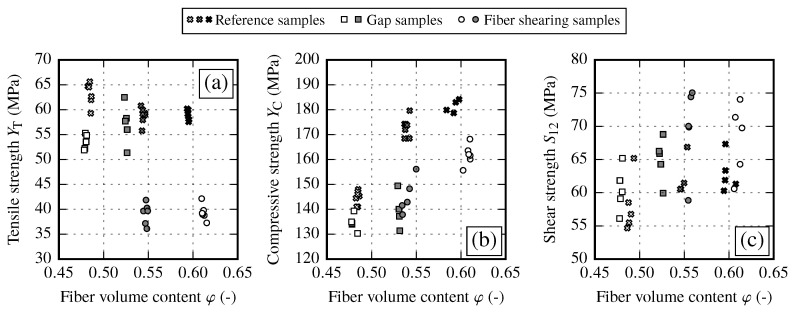
Comparison of the transverse strengths ($Y_{T}$ (**a**) and $Y_{C}$ (**b**)) and the in-plane shear strength $S_{12}$ (**c**) for different draping effect types.

By analyzing the different strength values of each draping effect no clear overall interrelation between the draping effects can be observed (cf. Figure 20). In general, the in-plane shear strength $S_{12}$ and the transverse compressive strength $Y_{C}$ show a trend towards higher values with increasing FVC. However, the data show a significant reduction of compressive strength $Y_{C}$ due to draping effects. On the other hand, the transverse tensile strength $Y_{T}$ seems to be defined by two clusters. For reference and gap samples the transverse tensile strength yields $Y_{T}\approx 58\;M\mathrm{Pa}$. Fiber shearing samples yield $Y_{T}\approx 40\;M\mathrm{Pa}$, thus having a clearly negative influence on the transverse tensile strength.

When comparing the samples with draping effects, the reference samples achieve the highest strength values for the transverse strength. In general fabric deformations resulting from transverse tension (gaps) or compression (pure shear) lead to lower transverse strength values. The in-plane shear strength is more diffused. However, the fiber shearing samples seem to achieve the highest values. As each strength value defines the shape of the failure envelope, the influence of the draping effects and the recommendations regarding necessary material characterization tests are discussed in the following sections.

### 5.2. Failure Envelope for Reference Samples

The determined increase of the material stiffness in different directions with increasing FVC corresponds to already known material behavior [29,30,31]. In addition to the fact that draping effects have an impact on the resulting material stiffness, to define the limitation of use, the strength values and the resulting failure envelopes are evaluated. As shown in Section 3.1 and Section 3.2 the resulting strength values vary depending on the actual draping effect. Each strength value is also affected by the FVC. While tensile strength in fiber direction can be estimated by a simple parallel-connected model of fiber and matrix, compressive strength is a result of the misalignment angle of the fabric [39,45]. A validated approach to determine the transverse tensile and compressive strength values as well as the in-plane shear strength values does not exists [23,51,52,53]. However, by utilizing the IFF criteria from Section 4.1, a failure envelope for each FVC can be determined. As the median strength values $Y_{T}$, $Y_{C}$ and $S_{12}$ are known from experimental results, the only free parameter is the inclination parameter *p*. Since this parameter defines the slope of the failure envelope in the $\sigma_{22}$-$\tau_{12}$ plane, only one additional supporting point is needed. For instance if an OAT45° test is performed the envelope in the $\sigma_{22}\geq 0$ quadrant can be defined (cf. Figure 3). However, this can lead to an overestimation of the failure envelope in the $\sigma_{22}<0$ quadrant. Therefore, additional support points are needed. In addition to the performed tests on reference samples, further OAC and off-axis tensions (OAT) tests have been conducted. Since the same material and also the same manufacturing process for reference samples have been used, the results of OAC50° tests are included to complete the failure envelope. The additional tests have a slightly different FVC φ≈{50%,55%,60%}. Nevertheless, it is assumed that this small difference of FVC does not significantly influence the resulting stresses.

**Figure 21:**
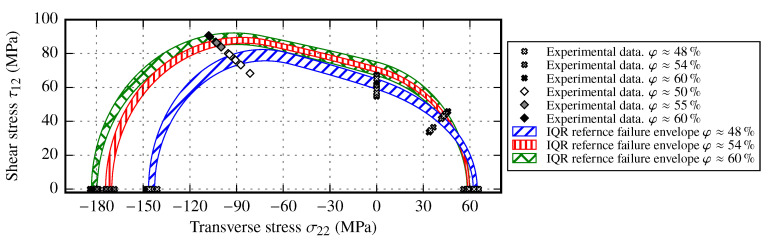
Resulting failure envelope ranges for reference samples at three different fiber volume contents.

By using the upper and lower quartiles of each strength value of reference samples, a range of the failure envelope for each FVC can be created. However, if the supporting points at OAT45° and OAC50° are compared to the predicted failure envelope, they always tend to be outside the predicted range. Therefore, each test result was reevaluated regarding the feasibility of the determined strength value. While the transverse strength values $Y_{T}$ and $Y_{C}$ undergo a uniform stress distribution and tend to fail by the specific failure mode, the ASTM 7078 in-plane shear tests utilize a notch to induce a specific failure mode. Due to this notch the strain and stress distribution is more complex and creates a multiaxial stress state near the notch. This indicates that the determined shear strength values are the lower bound of the actual shear strength range. By defining the shear strength $S_{12}$ and the inclination parameter *p* as free parameters of the IFF criteria, the derived failure envelope ranges for reference samples are given in Figure 21.

From the derived ranges it becomes obvious that the transverse stress has a major impact on the shape of the failure envelope. Since a significant increase of the transverse compressive strength with increasing FVC was observed, the failure envelope is enlarged in the $\sigma_{22}<0$ quadrant (cf. Section 3.1.2). This increase is also supported by the OAC50° experimental results. On the other hand, for positive transverse stresses the difference of the experimental results for each FVC is relatively small, while the scatter of each supporting point remains high. Therefore, the failure envelopes tend to overlap and suggest that no impact of the FVC on the failure envelope can be observed. The observed characteristic on the $\sigma_{22}>0$ axis correlates very well with a matrix dominated behavior under transverse tensile loads. Hence, it is important to perform experiments with negative transverse stresses, such as OAC45° or similar, rather than only perform tests with positive transverse stresses to get the complete picture of the failure envelope. Since the results of the in-plane shear strength based on the ASTM 7078 standard correspond to the lower bound of the actual shear strength range, it is recommended to perform either OAT and OAC tests near the shear stress axis (for instance OAC30° and OAT30°) and use these support points to fit an IFF criteria or use hoop wound samples to create a uniform stress distribution to get more reliable values for the shear strength. To further reduce the amount of necessary experimental tests to determine the failure envelope at different FVCs, it is suggested to perform the tests at φ≈[50%,60%] and interpolate linearly over these bounds to determine the other strength values.

### 5.3. Failure Envelope for Samples with Gaps

**Figure 22:**
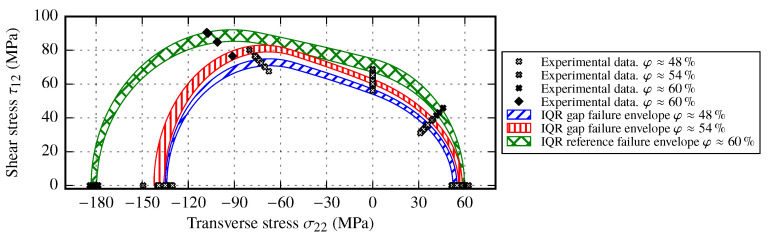
Resulting failure envelope ranges for samples with gaps at φ≈{48%,54%} compared to the reference samples failure envelope at φ≈60%.

By using the strength values of the samples with gaps (cf. Section 3.2.1), it is obvious that strength values are commonly reduced compared to the reference strength values. In fact, the transverse strength values $Y_{T}$ and especially $Y_{C}$ are highly affected. On the other hand, the in-plane shear strength seems to be increased if the strength values at each FVC are compared to the results of the reference samples. Additionally to the determined strength values, the support points based on the results of the OAT45° and OAC45° tests are used to fit the failure envelope for samples with gaps. By using the upper and lower quartiles of each strength value of samples with gaps, a range of the failure envelope for each FVC is created (see Figure 22).The resulting envelopes for gapping show that the bounds on the transverse stress axis are very similar. With decreasing FVC, the decrease of the transverse strength values of gapping samples is commensurately small compared to the reference samples with similar FVC. However, a clear distinction of the envelopes along the in-plane shear axis is observed. The condition that the shear strength range must be increased is confirmed by the OAT and OAC test results. This indicates that gaps in the fabric tend to reduce only the transverse strength values $Y_{T}$ and $Y_{C}$, while load bearing capabilities due to shear loads are not affected by gaps. Since the initial condition for samples with gaps is the reference with a FVC φ≈60%, the contrast between these envelopes is even more significant. All strength values of the reference samples are reduced compared to the samples with gaps. The overall difference of the resulting failure envelopes for the two different gapping sizes may result from the gaps itself rather than from the FVC alone. In case gaps should be considered in laminates, based on the observed results, it is recommended to perform at least one test series with a moderate gap width, which should correspond to the average FVC value (i.e., φ≈55%) of the composite part.

### 5.4. Failure Envelope for Fiber Shearing Samples

**Figure 23:**
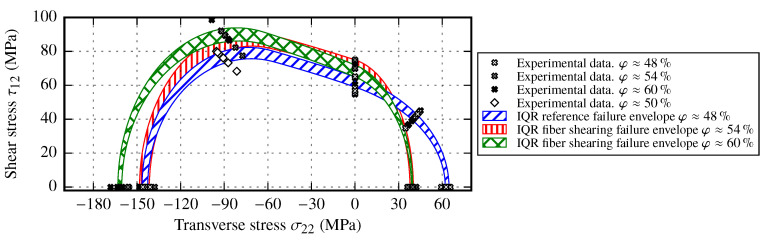
Failure envelope ranges for samples with an imposed fiber shearing at φ≈{54%,60%} in comparison to the reference samples envelope at φ≈48%.

One of the major deformation modes of the fabric used is simple shear. However, in corner regions pure shear is more likely. By utilizing the determined strength values from Section 3.2.2 and the additional OAT45° and OAC45° test results with imposed shearing, the failure envelopes for each shear angle α={27°,36.5°} is given in Figure 23.

By analyzing both failure envelopes of φ≈48% and φ≈54% two major observations can be made. First, both transverse strength values $Y_{T}$ and $Y_{C}$ are strongly affected by shearing of the fabric. Especially the transverse tensile strength $Y_{T}$ is reduced by about 33% compared to the strength resulting from reference samples. Second, the in-plane shear strength and the transverse strength ranges indicate to be independent of the imposed shear angle. The envelope ranges for both shear angles overlap from $\sigma_{22}=\left[-90\;M\mathrm{Pa},0\;M\mathrm{Pa}\right]$. By evaluating the maximum stresses of the OAT45° tests, one further aspect appears: the stress $\sigma_{22}^{OAT45}$ coincide with transverse tensile strength $Y_{T}$. This fact indicates that by applying shear to the fabric material, the resulting failure for $\sigma_{22}\geq 0$ is independent of the acting shear stress $\tau_{12}$ within the laminate. A possible explanation for this condition is the stitching of the fabric. By applying a pure shear mode to the fabric, the rovings are compressed against each other, while on the zigzag side of the fabric half of the stitching yarns are stretched and the other half forms out-of-plane undulations [5]. During the manufacturing process of the laminate, the stitching is pressed into the laminate and acts like a local imperfection, which causes a inhomogeneous stress distribution and therefore premature failure. On the other hand, the transverse compressive strength $Y_{C}$ increases towards higher shear angles or FVC. In contrast to reference samples, the increase is comparatively small and does not reach the same strength values. In conclusion, it can be said that the draping effect fiber shearing leads to a compression of the failure envelope along the transverse stress axis, without affecting the shear strength. It is therefore recommended to perform at least one set of transverse tensile tests on samples with imposed shear (e.g., α=30°) to determine the resulting tensile strength $Y_{T}$. Additionally, two test sets with different shear angles α seem to be suitable to determine the transverse compressive strength $Y_{C}$. The shear strength $S_{12}$ can be assumed to be the same as from the reference sample tests.

### 5.5. Comparison of the Impact of each Draping Effect on the Failure Envelope at the same Fiber Volume Content

**Figure 24:**
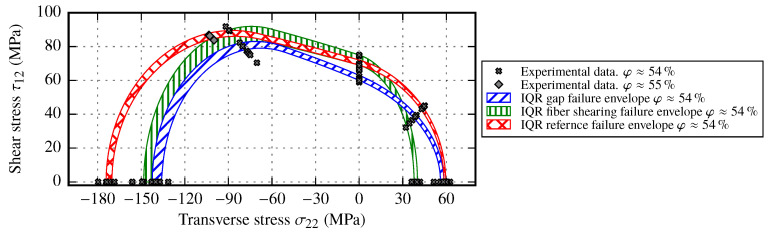
Comparison of the failure envelope ranges for reference samples, samples with gaps and samples with imposed shear at the same fiber volume content.

Each draping effect has its own impact on the failure envelope compared to itself. On the other hand, a clear distinction between the originate reference samples at a certain FVC and the resulting two different FVC failure envelopes can be observed. As the FVC at φ≈54% is common for all evaluated draping effects, the comparison of the resulting failure envelope ranges is given in Figure 24.

Although the FVC is the same in all samples, the resulting envelopes show a clear distinction. For instance the reference samples’ envelope, which is valid for local cavity height changes with almost no deformation of the fabric, enwraps the other both envelopes. This indicates that in-plane deformation of the fabric has a more drastic impact on the failure envelope than the resulting fiber volume content change. By analyzing the resulting strength evolution for different deformation types of the fabric, the following observations can be made:

- For transverse compressive strength $Y_{C}$, the highest decrease is caused by gaps. The draping effect fiber shearing leads to similar strength value as the ones resulting from gaps. Strength values of reference samples are about 25% higher.
- For transverse tensile strength $Y_{T}$, the clearest distinction which can be observed is the 33% drop of strength for samples with imposed shear. The draping effect gapping seems to have no negative effect on the transverse tensile strength.
- For in-plane shear strength $S_{12}$, only samples with gaps slightly reduce the strength, while other deformation modes lead to roughly the same shear strength.

If the shapes of the envelopes are compared at combined stress loads OAT45° and OAC45°, the impact on the combined failure stresses is smaller than on the pure transverse stresses. In conclusion, the different draping effects lead to failure envelopes which have similarities, but in the end they must be interpreted as individual “fabric types”. Nevertheless, according to the experimental results the used failure criteria can be used to determine the failure behavior for combined loads for each draping effect. In order to evaluate more in depth the impact of the FVC on each draping effect, the FVC should be also adjusted by the laminate thickness. Therefore, each deformation stage of each draping effect could be separately evaluated at different FVC to determine the corresponding strength values.

By comparing the resulting failure envelopes, guidelines to design composite parts can be made. The strength values of the reference samples create the largest failure envelope. It is followed by the fiber shearing and gap envelope (cf. Figure 24). If the failure behavior of all evaluated draping effects should be considered, than the failure envelope due to gaps should be used. However, the transverse tensile strength $Y_{T}$ due to gaps must be further reduced by a factor of ⅔ to consider the failure behavior of the effect fiber shearing. In this way, a conservative failure envelope is created which considers each evaluated draping effect. Since here a gap width of $w_{g}=1.1\;mm$ (which correspond to a FVC φ≈54%) is used to compare the different failure envelopes, the resulting failure envelope is even more shrunken, if the gap size is increased (cf. Figure 22). Further conclusions result from the reference samples (see Section 5.2) regarding the experimental effort to consider FVC-dependent strength. Therefore, for a conservative material characterization, it is recommended to determine the largest gap size after the draping process, create samples with the determined gap size at two different FVC and perform coupon tests to obtain the necessary strength values for the failure envelopes.

### 5.6. Resulting Waviness Stiffness and Strength Compared to Analytical Solutions

**Figure 25:**
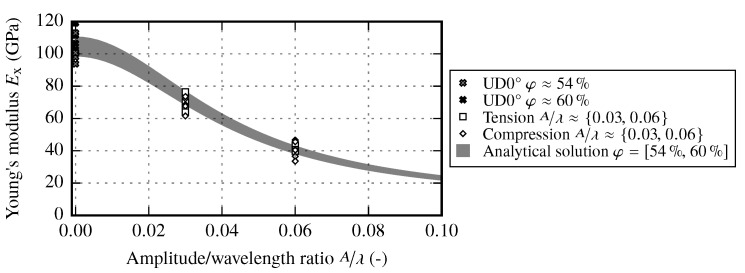
Comparison of the experimental results to the range of the analytical solution for the effective material stiffness $E_{x}$ in fiber direction, depending on the imposed amplitude to wavelength ratio.

In addition to the previously evaluated draping effects and their impact on the resulting IFF, waviness has an impact primarily on the loads in fiber direction. As shown in Section 4.2 the resulting stiffness can be determined from the basic material properties $E_{1}$,$E_{2}$,$G_{12}$ and $\nu_{12}$. By observing Equation (13) it is obvious that the effective stiffness $E_{x}$ yields $E_{1}$ in case of A/λ=0. A further observation can be made: besides the the local material stiffness $E_{1}$, the shear modulus $G_{12}$ has a significant impact on the resulting effective stiffness. Additionally, the imposed waviness has an effect on the resulting FVC that needs to be considered by analyzing the analytical solution (cf. Figure 18). The samples with waviness have FVCs comparable to the reference samples at φ≈54%. Therefore, the FVC for samples with waviness varies between 54% to 56%. As the material parameters of the reference samples are known at FVC φ≈54% and φ≈60%, the analytical solution is evaluated as a range of the effective stiffness $E_{x}$. As shown in Section 2.2, composites with carbon fibers require two parameters to determine the stiffness $E_{1}$ ($E_{1}^{\mathrm{init}}$ and $dE_{1}\;/\;d\varepsilon_{11}$). Since the actual fiber stiffness $E_{1}$ is a function of the strain $\varepsilon_{11}$, a direct comparison with the stiffness $E_{x}$ of wavy samples is difficult. However, for the sake of comparison, the static modulus $E_{1}^{\mathrm{init}}$ is used as input for the analytic solution at different FVCs. The resulting range is given in Figure 25.

The resulting range shows that the width of the effective modulus $E_{x}$ is reduced with increasing amplitude to wavelength ratio. Comparing the experimental results to the range of the analytical solutions show a very good agreement. In Figure 25 the results of the tensile and compressive tests are plotted. The loading direction has therefore no effect on the resulting stiffness.

**Figure 26:**
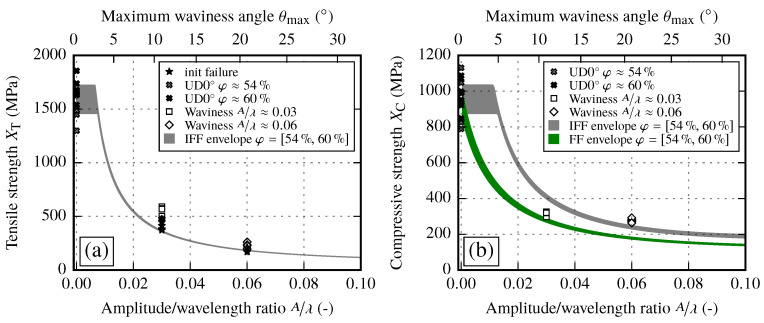
Comparison of the experimental results at different amplitude to wavelength ratios A/λ (or maximum waviness angle $\theta_{\max}$) with the analytical predictions for tensile (**a**) and compressive (**b**) tests.

On the other hand, to evaluate the analytical strength prediction is more complex. As described in Section 4.3, two separate approaches are used to analyze the resulting strength of samples with waviness. One results directly from the IFF criteria and the other is based on fiber failure. Similar to the analytical prediction of the effective stiffness, the strength is evaluated in a range of φ≈{54%,60%}. A strength range can be created by utilizing Equations (20) and (21) as well as the strength values of the reference samples. The resulting range for tensile and compressive tests is given in Figure 26.

By using the IFF criteria the resulting strength of waviness for small A/λ ratios (or $\theta_{\max}$ angles) yields the same tensile or compressive strength as non-undulated laminates. Further, the fiber failure criterion shows an instant reduction of the resulting strength for an increasing A/λ ratio. If the ranges of the analytical solutions are compared to the experimental results, several conclusions can be drawn for each load direction.

For tensile tests (Figure 26a), the IFF criteria coincide very well with the first occurrence of IFF cracks. However, the final failure strengths reach values which are 22% to 39% higher than the initial failure stresses. This condition is caused by crack propagation along the fiber orientation and cannot be captured by the IFF criteria. Therefore, the analytical solution defines a conservative solution for predicting failure strength values of samples with waviness.

For compression tests (Figure 26b), the resulting strength values at different A/λ ratios yield similar values. If the prediction of the strength range based on the IFF criteria is compared to experimental results, only at A/λ≈0.06 a good correlation can be observed while the strength at A/λ≈0.03 is overestimated. However, by adding the fiber failure criterion, the smaller waviness ratio correlates in very good agreement with the analytical solution. This leads to the conclusion that a transition from fiber dominant failure to an IFF is one of the possible explanations.

In conclusion, the prediction of the stiffness for tensile or compressive loads is quite accurate, if the material parameters for non-undulated laminates are known. The IFF criteria deliver a conservative estimation of the resulting tensile strength of laminates with waviness. Due to a very small strength range with increasing A/λ ratios, test series at only one FVC are needed. Since only positive $\sigma_{22}$ stresses occur at the maximum misalignment angle $\theta_{\max}$, the transverse tensile strength $Y_{T}$, the in-plane shear strength $S_{12}$ and the inclination parameter *p* are needed. To determine the strength of laminates with waviness for compressive loads, the same assumptions for the IFF failure as for the tensile loads can be made. Since the evaluated $\sigma_{22}$ stresses are moderate, the second case of Equation (5) can be utilized where only the in-plane shear strength $S_{12}$ and the inclination parameter *p* are necessary. Additionally, based on the experimental observations a transition from fiber failure to IFF occurs and the resulting strength of laminates with waviness can be estimated as following

(22) $$X_{C}=\left\{\begin{array}{cc} \frac{2X_{C}^{UD0{}^{\circ}}S_{12}}{2S_{12}\cos^{2}\theta_{\max}-X_{C}^{UD0{}^{\circ}}\sin2\theta_{\max}}, & \theta_{\max}<10{}^{\circ}\\ \min\{X_{C}{|}_{\theta_{\max}=10{}^{\circ}},\sigma_{\mathrm{xx}}^{\mathrm{IFF}}\}, & \theta_{\max}\geq 10{}^{\circ} \end{array}\right.$$

where $X_{C}^{UD0{}^{\circ}}$ correspond to the compressive strength of non-undulated laminates, $S_{12}$ is the in-plane shear strength, $X_{C}{|}_{\theta_{\max}=10{}^{\circ}}$ is the resulting strength at $\theta_{\max}=10{}^{\circ}$ from Equation (21) and $\sigma_{\mathrm{xx}}^{\mathrm{IFF}}$ is the solution of the IFF criteria at $f_{E}\overset{!}{=}1$.

## 6. Conclusions

The goal of this research was to investigate the impact of different draping effects in unidirectional non-crimp fabrics (UD-NCF) on the mechanical behavior of the cured continuous fiber reinforced plastic. The practical benefit of this study lies in the simultaneous investigation of changes in fiber volume content and fiber orientation due to draping effects. For that purpose, a comprehensive experimental test program with different predefined draping effects at different fiber volume contents (FVC) was performed. Two major aspects that occur during draping of UD-NCF were considered: deformation of the rovings and deformation of the stitching yarn. The stitching can be deformed by transverse normal or shear strains, leading to ***gaps*** between the rovings or to ***fiber shearing***. With compression in fiber direction or draping-induced run length differences of two adjoining rovings, local ***fiber waviness*** can occur. Since all draping effects have an impact on the resulting fiber volume content φ, the FVC was used as the common ground to compare the different deformation modes. A DOE with undeformed reference samples at φ≈{48%,54%,60%} and with samples including draping effects at corresponding fiber volume contents was defined and executed. The necessary preforms and samples were manufactured by previously developed tools to implement predefined gap sizes, fiber shearing and fiber waviness. Based on mechanical test results including full-field strain measurements, the impact of the draping effects on the mechanical properties and the failure behavior were analyzed. To evaluate the stiffness and failure behavior in fiber direction, the stiffness and strength of samples with waviness were compared to reference samples with straight fiber orientation. Properties perpendicular to the fiber direction were evaluated by analyzing the matrix-dominated failure behavior for combined loads. The corresponding inter-fiber failure envelopes according to the PUCK criterion were determined based on supporting points resulting from the different test series.

As expected, the resulting material stiffness increased with increasing fiber volume content (FVC), both in fiber direction and perpendicular to the fiber direction. This stiffness effect was found to be more or less independent of the cause of FVC variation, induced by either thickness change, gaps or fiber shearing.

With regard to the strength values in transverse fiber direction, the results are divergent. For each draping effect, the matrix-dominated strength values such as transverse tensile and compressive strength as well as in-plane shear strength yield different results for equal FVC:

- For undeformed reference samples, a significant increase of the transverse compressive strength with increasing FVC was observed. Transverse tensile strength and shear strength were not affected by varying FVC, or just to a small degree.
- Samples with gaps reduce the transverse compressive strength significantly, independently of the gap size and the resulting FVC. On the other hand, shear and transverse tensile strength are hardly affected by gapping.
- Contrary to the reference and gap test results, the effect fiber shearing reduces both the transverse compressive and the transverse tensile strength significantly to a constant value, independent of the present FVC.

By comparing the failure envelopes for inter fiber failure, the following observations could be made:

- For undeformed reference samples, the envelopes are enlarged with increasing fiber volume content towards higher transverse compressive and in-plane shear strength values, while the transverse tensile strength remains constant.
- For samples with gaps, the failure envelope is compressed to smaller compressive strength, while the shear and tensile strength remain almost unaffected.
- In contrast, the draping effect fiber shearing compresses the failure envelope on both ends of the transverse stress axis, reducing transverse compressive as well as tensile strength, without significant change of the shear strength.

Comparing the failure envelopes of different effects at the same FVC leads to the conclusion that, generally speaking, ***each draping effect should be treated as a different fabric type regarding its failure behavior***, rather than having just the FVC as the common ground.

Evaluating the failure behavior in fiber direction, in accordance with other previous publications, the strength values increase due to increasing fiber volume content. However, in-plane ***fiber waviness*** significantly affects the material parameters. The compressive properties are reduced more severely than the tensile properties. In this study it was found, the governing failure modes depend on the load direction. Under tensile loading in fiber direction, a reorientation of the fibers is observed, accompanied by initial crack formation due to inter-fiber failure, before final fiber failure occurs. With the developed analytical model, the initial tensile failure can be predicted accurately by utilizing the PUCK criteria. Under compressive loading in fiber direction, a transition from a fiber dominated failure to an inter-fiber failure was found for an increasing misalignment angle that can be predicted as well.

The findings presented in this paper are specific for the evaluated composites using dry UD-NCF fabric or any other fabric with similar textile architecture. The results of this study are an addition to the previously evaluated draping effects that mainly exist for prepregs with different deformation mechanisms. A whole experimental database of material parameters for this representative UD-NCF with draping effects is provided. Further added value lies in the detailed mechanical characterization of the draping effects gapping and fiber shearing. The results are of great importance in the holistic design of fiber composite components using the virtual process chain. Using draping simulation, draping effects can be predicted and quantified. After mapping these effects to a structural simulation, a more realistic prediction of the structural loads and failure behavior can be achieved.

In the case that occurring draping effects should be considered within a structural simulation, the following recommendations are given to determine the necessary material properties: To keep the number of mechanical tests reasonably low, it is suggested to test samples at two FVCs, a low and high one, and to interpolate linearly in-between. OAC45° and OAT45° tests are recommended to obtain reliable supporting points for the inter-fiber failure envelopes. To determine the in-plane shear strength, it is recommended to perform an off-axis compression or off-axis tension test with high local shear stresses rather than a pure shear test, since V-notch-rail tests deliver lower bound strength values. To consider all evaluated draping effects by one failure envelope, it is recommended to use the strength results of samples with a specific gap size to be at the conservative side. However, the determined transverse tensile strength must be further reduced by a factor of ⅔ in order to consider the impact of fiber shearing effect. If possible, a suitable gap size can be determined by analyzing the maximum gap size occurrence in a final composite part preform. In conjunction with the strength results at two different fiber volume contents, such approach provides a conservative design method which considers inter-fiber failure for different draping effects.

## Figures and Tables

**Table 1:** Materials.

Type	Manufacturer	Specification
Unidirectional carbon fiber fabric	Zoltek	PX35, 50 K, 338 g m^−2^
Non-reactive pre-applied binder	Huntsman	XB3366
Reactive powder binder	Huntsman	XB6087
Epoxy resin	Sika	CR170/CH150-3
Internal mold release agent	Würtz	PAT 657 BW

**Table 2:** Design of experiments in transverse fiber direction.

Parameters	FVC	Reference (Plate Thickness)	Gapping (Gap Size)	Transverse Compression (Shear Angle)
Young’s modulus $E_{2}$ Shear Modulus $G_{12}$ Tensile strength $Y_{T}$ Compressive strength $Y_{C}$ Shear strength $S_{12}$ Tensile stress $\sigma_{22}^{OAT45}$ ^1^ Shear stress $\tau_{12}^{OAT45}$ Tensile stress ^2^ $\sigma_{22}^{OAC45}$ Shear stress ^2^ $\tau_{12}^{OAC45}$	φ≈48% φ≈54% φ≈60%	2.25 m m 2.00 m m 1.80 m m	3.3 mm 1.2 mm /	/ 27.0° 36.5°

^1^ see list of abbreviations. ^2^ not for reference samples.

**Table 3:** Design of experiments in fiber direction.

Parameters	FVC	Reference (Plate Thickness)	Waviness (Amplitude/wavelength Ratio)
Young’s modulus $E_{1}$ Slope ^*^ $dE_{1}\;/\;d\varepsilon_{11}$ Poisson’s ratio ^∗^ $\nu_{12}$ Tensile strength $X_{T}$ Tensile strength $X_{C}$	φ≈48% φ≈54% φ≈60%	2.25 m m 2.00 m m 1.80 m m	/ ≈0.03 and 0.06 /

* not for waviness.

**Table 4:** Tooling and ply arrangements of implemented draping effects [5].

Effect	Principle of Specimen Preparation	Ply	Stacking
Reference		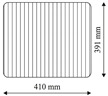	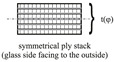
Gapping	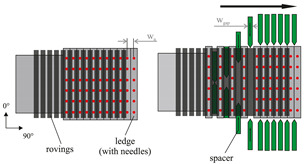	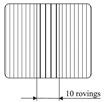	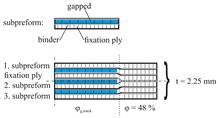
Fiber Shearing	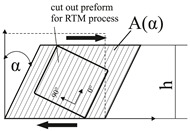	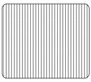	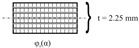
Waviness	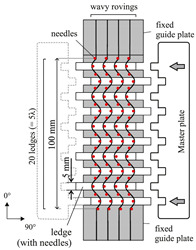	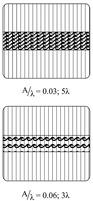	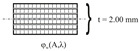

**Table 5:** RTM-process parameters.

Process Parameter	Value
Mix ratio by weight-resin:hardener	100:24
Percentage of internal release agent on total resin weight	1.6%
Resin temperature-resin/hardener	≈65 ${}^{{}^{\circ}}C$/≈32 ${}^{{}^{\circ}}C$
Mix head pressure-resin/hardener	≈130 bar/130 bar
Tool temperature	≈100 ${}^{{}^{\circ}}C$
residual cavity pressure before injection (vacuum)	1.3 mbar to 2.0 mbar
evacuation time	2 min
resin flow rate	10 g m^−1^
Cavity pressure	50 bar to 60 bar
Curing time	13 min
Post cure	4 h @ 140 ${}^{{}^{\circ}}C$

**Table 6:** Standards and specimen dimensions for material testing in transverse fiber direction of reference samples, samples with gaps and fiber shearing samples.

Load Direction	Standard	Width (mm)	Length (mm)	Tab Length/ Free Length (mm)	Testing Machine/ Load Cell	Grips
Tension ^1,2^	ISO 527-5, type B	25	300	75/150	Zwick Z100/ 100 kN	hydraulic
Off-axis tension ^1^	ISO 527-5, type B	25	300 (200) ^3^	75 (25) ^3^ /150	Zwick Z100/ 100 kN	hydraulic
Shear ^1^	ASTM 7078	76	56	/	Zwick 1475/ 250 kN	V-notch shear fixture
Off-axis compression ^1^	ISO 14126	10	140	65/10	Zwick 1475/ 100 kN	HCCF ^4^
Compression ^5^	ISO 14126	10	140	65/10	Zwick 1475/ 100 kN	HCCF ^4^

^1^ Digital image correlation with Aramis from GOM. ^2^ Video extensometer strain measurement for reference samples. ^3^ Values in brackets for samples with draping effects. ^4^ Hydraulic Composite Compression Fixture. ^5^ Laser extensometer strain measurement for reference samples.

**Table 7:** Standards and specimen dimensions for material testing in fiber direction of reference samples and samples with imposed waviness.

Load Direction	Standard	Width (mm)	Length (mm)	Tab Length/ Free Length (mm)	Testing Machine/ Load Cell	Grips
**Reference**						
Tension ^1^	ISO 527-5, type A	15	300	75/150	Zwick Z100/ 100 kN	hydraulic
Compression ^2^	ISO 14126	10	140	65/10	Zwick 1475/ 100 kN	HCCF ^3^
**Waviness**						
Tension ^4^	ISO 527-5, type A	25	300	75/150	Zwick Z100/ 100 kN	hydraulic
Compression ^5^	ISO 14126	17	115	37.5/40 ^6^	Zwick 1475/ 100 kN	HCCF ^3^

^1^ Video extensometer strain measurement for reference samples. ^2^ Laser extensometer strain measurement for reference samples. ^3^ Hydraulic Composite Compression Fixture. ^4^ Digital image correlation with Aramis from GOM. ^5^ Strain measurement on the edge of the sample (with Aramis from GOM). ^6^ With support block to prevent premature buckling.

**Table 8:** Reference samples—material stiffness properties at different fiber volume content values (median values and interquartile range (IQR) in brackets).

Fiber Volume Content	φ≈48%	φ≈54%	φ≈60%
Young’ s modulus $E_{1}^{\mathrm{init}}$ (GPa)	89.1 (6.42)	101.0 (2.50)	104.6 (9.30)
Young’ s modulus $E_{2}$ (GPa)	7.0 (0.07)	7.1 (0.20)	8.0 (0.09)
Shear modulus $G_{12}$ (GPa)	3.2 (0.10)	3.5 (0.19)	3.9 (0.03)
Poisson’ s ratio $\nu_{12}$ (-)	0.34 (0.04)	0.32 (0.03)	0.33 (0.01)
Slope $dE_{1}\;/\;d\varepsilon_{11}$ (TPa)	1.04 (0.20)	1.30 (0.11)	1.18 (0.16)

**Table 9:** Reference samples—material strength at different fiber volume contents (median values and interquartile range (IQR) in brackets).

Fiber Volume Content	φ≈48%	φ≈54%	φ≈60%
Tensile strength $X_{T}$ (MPa)	1251 (35.4)	1500 (140.8)	1670 (79.9)
Compressive strength $X_{C}$ (MPa)	836 (40.3)	989 (145.0)	939 (114.3)
Tensile strength $Y_{T}$ (MPa)	64 (2.5)	59 (1.2)	59 (1.6)
Compressive strength $Y_{C}$ (MPa)	145 (3.8)	174 (4.0)	181 (3.7)
Shear strength $S_{12}$ (MPa)	57 (3.0)	61 (2.3)	62 (2.0)

**Table 10:** Gapping samples—summary of material stiffness results for samples with imposed gaps: From maximal gap width (φ≈48%) to initial state (φ≈60%).

Fiber Volume Content	φ≈48%	φ≈54%	φ≈60% (Reference)
Young’ s modulus $E_{2}$ (GPa)	6.3 (0.04)	7.5 (0.07)	8.0 (0.09)
Shear modulus $G_{12}$ (GPa)	3.0 (0.04)	3.6 (0.13)	3.9 (0.03)

**Table 11:** Samples with gaps—resulting material strength properties: From maximal gap width (φ≈48%) to initial state (φ≈60%).

Fiber Volume Content	φ≈48%	φ≈54%	φ≈60% (Reference)
Tensile strength $Y_{T}$ (MPa)	54 (2.6)	58 (2.3)	59 (1.6)
Compressive strength $Y_{C}$ (MPa)	134 (1.0)	139 (6.7)	181 (3.7)
Shear strength $S_{12}$ (MPa)	60 (2.8)	66 (2.0)	62 (2.0)

**Table 12:** Fiber shearing—material properties at different fiber volume contents (median values and IQR in brackets).

Fiber Volume Content	φ≈48% (Reference)	φ≈54%	φ≈60%
Young’ s modulus $E_{2}$ (GPa)	7.0 (0.07)	7.3 (0.06)	7.7 (0.09)
Shear modulus $G_{12}$ (GPa)	3.2 (0.10)	4.2 (0.07)	4.6 (0.48)

**Table 13:** Fiber shearing samples—summary of material strength from the initial state (φ≈48%) to different deformation states due to fiber shearing (φ≈54% and φ≈60%).

Fiber Volume Content	φ≈48% (Reference)	φ≈54%	φ≈60%
Tensile strength $Y_{T}$ (MPa)	64 (2.5)	40 (2.3)	39 (0.8)
Compressive strength $Y_{C}$ (MPa)	145 (3.3)	143 (6.7)	162 (2.7)
Shear strength $S_{12}$ (MPa)	57 (3.0)	70 (4.6)	70 (7.1)

**Table 14:** Experimental material properties of samples with waviness in different loading directions (all values are medians with the corresponding interquartile range in the brackets).

Amplitude/Wavelength Ratio	A/λ=0	A/λ≈0.03	A/λ≈0.06
Young’ s modulus $E_{x}$ (GPa)	101.0 (2.50)	70.0 (9.88)	42.0 (2.70)
Tensile strength $X_{T}$ (MPa)	1500 (140.8)	569 (72.4)	231 (20.5)
Compressive strength $X_{C}$ (MPa)	989 (145.0)	308 (17.9)	278 (10.1)
Initial tensile failure * (MPa)	-	413 (67.0)	190 (29.0)

* Approximate value readings based on visible occurring cracks.

## Abbreviations

The following abbreviations are used in this manuscript:

CoFRP	Continuous fiber reinforced plastic
CFRP	Carbon fiber reinforced plastic
DOE	Design Of Experiments
FRP	Fiber reinforced plastic
FVC	Fiber volume content
IFF	Inter fiber failure
NCF	Non-crimp fabric
OACxx°	Off-axis compression at angle xx°
OATxx°	Off-axis tension at angle xx°
RTM	Resin transfer molding
UD	Unidirectional
